# *N*-Methylated Nucleobases Crystal Structures and π-π Stacking Interactions

**DOI:** 10.3390/molecules31081326

**Published:** 2026-04-17

**Authors:** Riccardo Cameli Manzo, Volodymyr Baran, Artem Shevchenko, Anastasia Sleptsova, Frank Hoffmann, Tomislav Stolar, Robert E. Dinnebier, Martin Etter

**Affiliations:** 1 Deutsches Elektronen-Synchrotron DESY, Notkestrasse 85, 22607 Hamburg, Germany; 2 Max Planck Institute for Solid State Research, Heisenbergstrasse 1, 70569 Stuttgart, Germany; 3 Institute of Inorganic and Applied Chemistry, Universität Hamburg, Martin-Luther-King-Platz 6, 20146 Hamburg, Germany; 4 BAM Federal Institute for Materials Research and Testing, Richard-Willstätter-Strasse 11, 12489 Berlin, Germany

**Keywords:** XRPD, SC-XRD, synchrotron-XRPD, methyladenines, methylguanines, nucleobases, solid-state, π-π stacking

## Abstract

Solid-state studies evaluating intermolecular geometries in methylated nucleobases are not extensively explored. In the course of the present study, we have solved the crystal structures of 1-, 3- and 7-methylated adenines and guanines, including the monohydrate and sesquihydrate forms of 3-methyladenine and 3-methylguanine, respectively, by means of single-crystal X-ray diffraction and synchrotron/laboratory X-ray powder diffraction (XRPD). In situ high temperature XRPD experiments, coupled with differential thermal analysis/thermogravimetry (DTA/TG) measurements, allowed for monitoring crystallographic changes after water removal of *N*3-methylated compounds, and the discovery of a high temperature polymorph in the case of 3-methyladenine. Our findings indicate that H-bonding schemes describe ribbon planar motifs of molecules in the majority of cases, or linear double-bonded strands of molecules in a few cases. π-π stacking interactions were compared with existing findings of theoretical calculations and existing crystallographic data, showing how *N*-methylated purine bases follow the trend predicted by Hunter and Sanders, 1990. The present study provides the first systematic experimental insights into the solid state of the presented compounds.

## 1. Introduction

Methylation represents an important epigenetic modification of nucleic acids, involved in gene regulation [1,2]. Exposure to cigarette smoke or pathological conditions such as tumors and immunodeficiencies can result in abnormal nucleobase alkylation, compromising DNA stability and replication [3,4,5,6]. Methylated nucleobases can then be found in urine as an indicator of whole-body turnover and degradation of methylated RNA [7,8]. In addition, methylated nucleobases are seeing a rising importance concerning studies in mechanochemistry [9,10], and tested for their potential anticancer activity [11,12]. Moreover, solid-state investigations provide experimental insights that can be applied to biological systems. In fact, we noticed that despite significant efforts in understanding the solution and gas-phase behavior of methylated nucleobases [13,14,15,16,17,18], there is still a surprisingly high lack of solid-state studies. Crystal structures of *N*-methylated nucleobases, with methylation on the rings, not on the functional groups (oxo and amino/imino) were solved only in the cases of 9-methylguanine (9mG), 9-methyladenine (9mA) and 3-methylguanine (3mG) sesquihydrate [9,19,20]. However, in the case of 3mG sesquihydrate, no atomic coordinates were available. Crystal structures of pure guanine and adenine were instead solved relatively recently [21,22].

Studies are reporting a growing interest in the description of noncovalent interactions of nucleobases for their application in material sciences [23,24,25], as well as their importance in stabilizing nucleic acids [26,27]. In fact, stacking between nucleobases is considered as one of the two factors that are mainly responsible for the stability of the DNA double helix, together with hydrogen bonding [28,29,30]. Furthermore, stacking interactions are specifically inferred to play a pivotal role in governing the selectivity of enzymes for removing damaged nucleobases in DNA due to alkylation [13] and the effect of post-transcriptional methylation in RNA [14].

A model for describing electronic interactions in aromatic systems accounting for the charge distribution was first proposed in a pioneering study by Hunter and Sanders, 1990 [31]. This model describes π-π stacking geometries in benzene rings as the result of the interplay between electrostatic effects and van der Waals (vdW) interactions. As a result of the balance between attraction and repulsion due to these interactions, aromatic rings can orient in different geometries, including parallel, parallel displaced, or T-shaped stacking, depending on the relative position between the aromatic rings and polarization effects on the π-systems. Schramm et al., 2025 [32] explained how, in parallel displaced benzene dimers, vdW interactions control the lateral displacement, whereas electrostatic effects are responsible for minimizing the distances between the two interacting rings at parallel displaced geometries [33]. Among π-π interactions, T-shaped stacking between nucleobases and aromatic amino acids can provide stability to many different enzymatic systems, including those involved in DNA transcription, replication, and repair [34]. Moreover, other types of π- interactions can occur involving an aromatic ring and ions or functional groups, such as XH-π, cation-π, anion-π or lone pair-π [35].

Simplistic experimental studies of π-π investigations on purine nucleobases are, to date, not fully explored. A study on pure 2-deoxyadenosine [36] has revealed its crystal structure; however, no stacking interactions were identified at that time. However, considering the fact that the study of Hunter and Sanders provided new insights into the definition and importance of π-π interactions for biological molecules only a few years later in 1990, the revisiting of findings prior to this study might be suggested. We note that the 2-deoxyguanosine crystal structure remains unknown as of today. Similarly, in the case of RNA bases bonded with ribose, the adenosine crystal structure is solved [37] while guanosine was not found in databases.

In the present study, we will report about the first solid-state experimental insights into 1-, 3- and 7-methylated adenines and guanines. The crystal structures were solved from synchrotron and laboratory X-ray powder diffraction (XRPD) data, and from single crystal X-ray diffraction (SC-XRD) data, where recrystallization to single crystals was required due to poor powder quality. Furthermore, in the case of 3-methyladenine and 3-methylguanine, crystal structures with molecular water, anhydrous crystal structures and polymorphism will be discussed following in situ high-temperature XRPD experiments. Packing motifs will be described according to H-bonding schemes. π-π stacking geometry parameters, particularly horizontal and vertical displacements, and relative orientations of stacked nucleobases will also be discussed, combining an overview of existing theoretical studies and existing crystallographic data on pure and N9-methylated guanines and adenines. The Hunter and Sanders equation will be used for modeling our experimental data, providing reasonable agreement with their predictions.

## 2. Results

In the course of this study, we have solved the crystal structure of 1-, 3- and 7-methylated purine nucleobases from SC-XRD and XRPD from laboratory and synchrotron data. Concerning XRPD analyses, most powders were investigated as received from the manufacturer. Exceptions consist of 1-methylguanine (1mG) and 3mG sesquihydrate which have been recrystallized in solution due to lack of crystallinity, and then solved with SC-XRD, and 3mG and 3-methyladenine (3mA) after thermal treatments. Table 1 summarizes relevant crystallographic information of the *N*-methylated nucleobases solved herein. All crystal structures, except 3mG sesquihydrate, were previously unknown. Notably, atomic coordinates for the 3mG compound could not be found in the original deposition [20]; we have thus solved the corresponding crystal structure anew. Thermal studies, including differential thermal analysis and thermogravimetric analysis (DTA-TGA) for 3mG, and in situ high-T XRPD have been carried out in order to remove water molecules from the solid-state structures of 3mA and 3mG. Particular attention has been paid to the analysis of the geometric parameters describing the stacking interactions of molecules, given their paramount importance in supramolecular chemistry. The definitions of the geometric parameters considered in this study are reported in Section 4.

### 2.1. 1-Methylguanine

**Figure 1:**
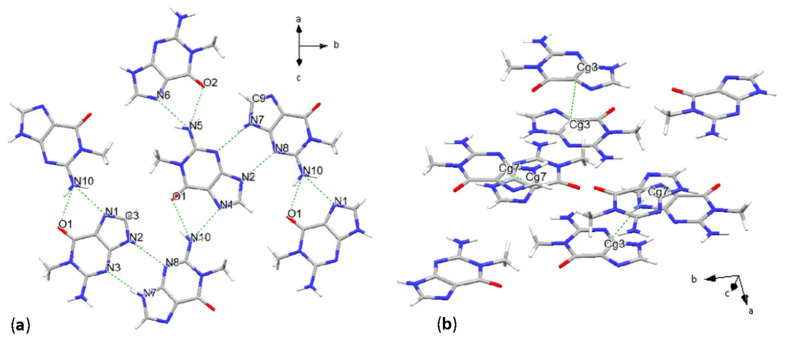
Crystal structure of 1-methylguanine. H-bonding scheme (**a**) and main stacking geometries (**b**).

Crystal structure solution from SC-XRD resulted in a monoclinic unit cell with the asymmetric unit consisting of two molecules, where the molecular arrangement is described by space group *P*2_1_/*c* [No. 14]. Intermolecular contacts are characterized by N-H···N, C-H···N and N-H···O hydrogen bonds (Figure 1a and Table 2). N5(amino) acts as a proton donor, forming bonds with the N6 and O2(oxo) sites, with donor-acceptor bond lengths (D···A) of 2.822(2) Å and 2.893(2) Å, respectively. N3 binds with the second molecule part of the asymmetric unit, forming an H-bond accepting a proton from N7, with D···A of 2.843(2) Å. Lastly, the N2···N8 distance is 2.844(2) Å. Weaker C-H···O bonds form, as in the case of C9, acting as a proton donor and O2, with a distance of 3.257(2) Å. Additional H-bonds generated by symmetry are reported in the Supplementary Materials (Table S1). These intermolecular interactions form a planar network stacking along the *a* + *c* crystallographic axes.

Concerning the stacking interactions, centroids were defined for pyrimidine (6-membered), imidazole (5-membered) and purine (9-membered) rings. Geometric parameters, referring to parallel displaced π-π stacking, are summarized in the latter cases, together with the donor–acceptor intermolecular interactions in Table 2.

Centroid distances vary from 3.4595(9) Å up to 5.2103(8) Å between 9-membered rings, 3.74 Å up to 5.86 Å considering the case of stacking of 5-membered ring centroids, and 4.1950(11) Å up to 4.7918(11) Å for 5-membered rings. Interplanar distances vary between 3.2229(7) Å and 3.3091(6) Å, while the β angle changes from a minimum of 16.7° for stacking of centroids defined as Cg3 (see Table 2), reaching a maximum value of 56.2° for a configuration of pyrimidine centroids (Table S2). The dihedral angle α ranges from 0° to 3.33°, indicating a high co-planarity of the molecules. Horizontal displacements range from 0.99 Å to 4.87 Å. Purine rings are stacking both with inverted and direct configurations, almost reaching a perpendicular orientation, with a value of 82.6° in the direct conformation, in the case of the stacking between centroids defined as Cg3 and Cg7 (Figure 1b, Table 2). Molecules assume a trans conformation in both cases, with the glycosidic sites in opposite directions. In the last case, Cg7 centroids stack with each other also in another direct, trans, conformation with a rotation angle of 79.8(3)°. In this case, the β angle reaches 52.1°, and the Cg-Cg distances are 5.2103(8) Å.

### 2.2. 3-Methylguanine Sesquihydrate

**Figure 2:**
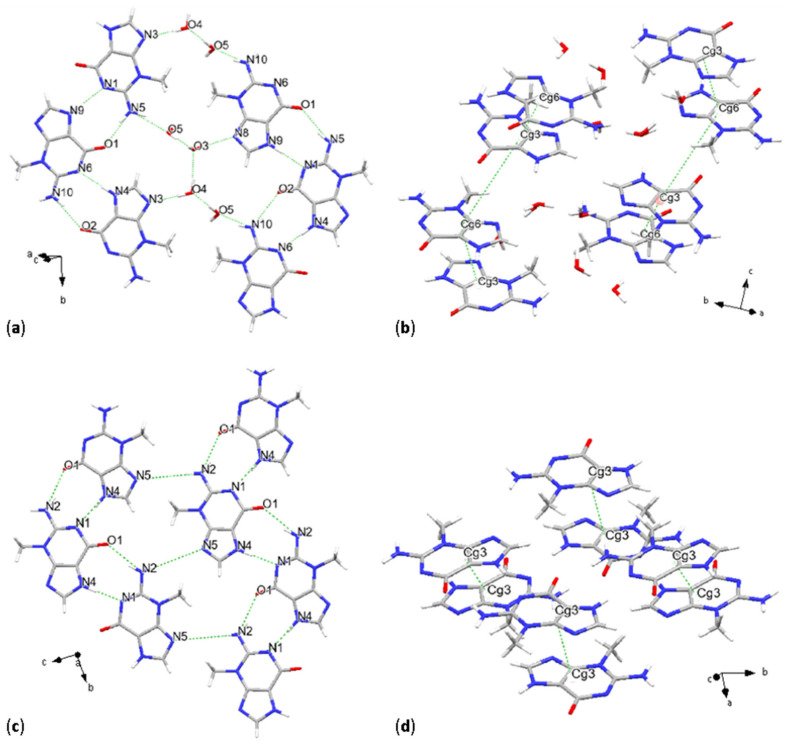
Crystal structures of 3-methylguanine sesquihydrate and 3-methylguanine. H-bonding schemes (**a**,**c**) and main stacking geometries (**b**,**d**).

Jaime E. Abola et al., 1976 [20] originally determined the crystal structure of 3mG sesquihydrate grown in aqueous solution by SC-XRD combining direct and Fourier methods. Here we have determined the crystal structure by SC-XRD at 100 K anew, resulting in a monoclinic unit cell, space group *P*2_1_/*c* [No. 14] and provided the so far missing atomic coordinates. The asymmetric unit consists of two molecules of 3mG and three molecules of water, O3, O4 and O5. Intermolecular interactions are represented by N-H···N, N-H···O and O-H···O. Molecular chains of 3mG are connected with water through hydrogen donor-acceptor bonds, forming planes that stack along the *c-a* crystallographic axes (Figure 2a). N(amino) acts as a proton donor, forming bonds with the oxo functional group and with water. One example of this bond is given by N10-H10A···O5(water) and N10-H10B···O2(oxo), corresponding values are reported in Table 3. Other shorter donor-acceptor bonds consist of N9-H9···N1, and O3-H3A···N8 and involving only H-bonds between water molecules O3-H3B···O2 and O3-H3D···O3.

Geometrical parameters, summarized also in Table 3, consist of a distance among centroids from 3.4578(4) Å to 5.7300(4) Å, for 9-membered fused rings according to symmetry translations and 3.4304(6)–5.6912(6) Å and 4.3927(5)–5.7554(5) Å for 5-membered and 6-membered rings, respectively. As in all the other cases, most of the analysis of this study was focused on pyrimidine + imidazole fused rings, and the different geometrical parameters of their π-π stacking interactions, consisting of parallel displaced rings, are as follows: interplanar distances are of 3.2409(3) Å and 3.3377(3) Å, and the 9-centroids defined β angles of 19.6° and 57.8°, and were horizontally displaced of 1.16–4.851 Å (Figure 2b). Dihedral angle values, 3.54(3)° and 4.49(3)° for the symmetry-generated pairs, indicate not fully planarly configured purine bases. Stacking configurations of these pairs are inverted, with a rotational angle of 159.85(5)°, and direct, with an angle of 10.6(2)°, and the glycosidic site is configured in cis and trans, respectively.

In order to evaluate H-bond geometry and stacking geometries for 3-methylguanine as a pure compound, the single crystals obtained by recrystallization from solution were ground and the obtained crystalline powder was used for in situ high-temperature XRPD experiments to monitor crystallographic changes until the expected crystalline water release.

**Figure 3:**
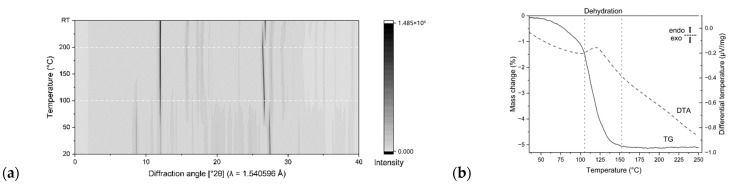
Thermal behavior of 3-methylguanine sesquihydrate. Contour plot showing diffraction pattern changes upon heating, with major crystal structure changes starting to occur from 100 °C (**a**), and thermogravimetric and differential thermal analysis (**b**).

Upon heating, we have discovered that significant changes in the crystal structure start to occur from 100 °C, with no further changes at 150 °C and 200 °C (Figure 3a). Further thermal analyses with DTA and TG were carried out on the as-purchased powders, revealing the onset of an endothermic process with onset at 100 °C and completion at 150 °C, consistent with XRPD analysis and observed as a heat intake, a consequence of water loss (Figure 3b). Molecular weight calculations from 3-mG sesquihydrate C_6_H_7_N_5_O-1.5(H_2_O), 191 g/mol, to the anhydrous C_6_H_7_N_5_O, 165 g/mol indicate an expected loss of 13 wt. % following a water release. The mass loss of 5% observed in our TG data is inconsistent with a sesquihydrate form; instead, TGA rather suggests a hemihydrate: C_6_H_7_N_5_O-0.5(H_2_O), 174 g/mol. Given the duality of the experiments, from crystals grown in solution for SC-XRD and from powders as purchased for DTA-TGA, we infer that the as-purchased compound has poor crystallinity. However, the ratio between water molecules and methylated nucleobase is lower, and in the recrystallization experiments, the ratio increases, forming the sesquihydrate.

### 2.3. 3-Methylguanine

Following the XRPD thermal analyses, crystal structure solution from synchrotron XRPD data collected at room temperature revealed planar configured molecules, whose arrangement is described by space group *P*2_1_/*c* [No. 14], similar to the case of 3-methylguanine sesquihydrate. The stacking sequence of the planes defined by the molecules is approximately along the crystallographic *a*-axis. The planar intermolecular interactions consist of N-H···N and N-H···O, following the same bonding scheme as it was the case of 3-methylguanine sesquihydrate: N(amino) is forming a bond with the oxo group of adjacent molecules, and N4-H3···N1, which in the previous case was labeled as N9-H9···N1 but still persisting in the same molecular sites (Figure 2c). After water removal, the second acceptor for amino group protons, which previously consisted of a water molecule, is now the site labeled as N5. This interaction resulted in the weakly bonded N2-H2···N5 with a D···A bond distance of 4.18(2) Å.

Parallel displaced π-π stacking occurs between purine bases, here focusing on 9-membered fused rings that form two different types of pairings, with centroid distances of 3.604(11) Å and 3.465(11) Å, β angles of 23.4° and 18.4°, and slippage of 1.434 Å and 1.094 Å. The rotation angle indicates a fully inverted configuration, while the position of the glycosidic site indicates a trans molecular conformation. The dihedral angle α was found to be 0°, indicating full planarity of the molecules relative to one another (Figure 2d).

### 2.4. 7-Methylguanine

Crystal structure solution from synchrotron XRPD data revealed that 7-methylguanine (7mG) molecules are stacking along the *b* and *a*–*b* axial directions and forming four chains within the unit cell. This arrangement is described by *P*$\bar{1}$ [No. 2] space group symmetry. The asymmetric unit consists of four molecules; two examples of 9-membered centroids are Cg15 and Cg16, defined as in Table 4.

**Figure 4:**
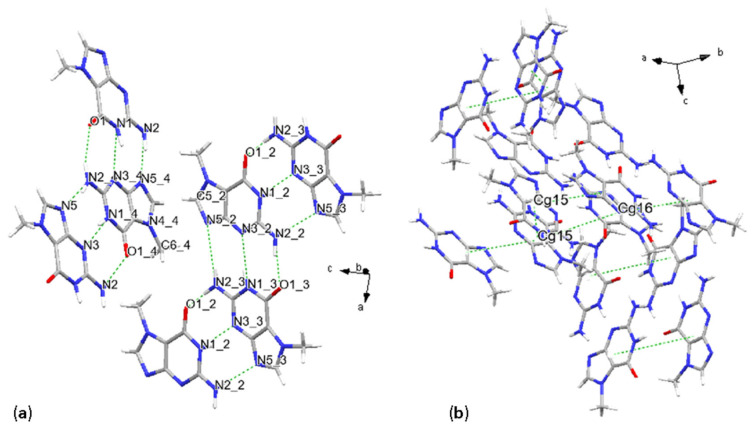
Crystal structure of 7-methylguanine. Main hydrogen bonding scheme, with representation of the molecular chains (**a**), and stacking geometries (**b**).

The H-bonding is the primary interaction involving these planarly configured chains of molecules; these interactions consist of N-H···N, N-H···O and C-H···O. Stronger H-bonds form three main different donor-acceptor schemes, which are then repeated four times coherently with the number of molecules per asymmetric unit: distances are N1_3-H3_3···N3_2 with 3.0(2) Å, N2_2-H2_2···O1_3 with 2.8(2) Å, and N2_2-H1_2···N5_3 with 3.35(18) Å as shown in Figure 4a and Table 4. Given the number of molecules in the asymmetric unit, the uncertainty of the result is higher due to the higher number of degrees of freedom in the modeling, as the crystal symmetry is lower than in the other nucleobase crystal structures. As previously mentioned in this study, the primary stacking interaction consisted of parallel displaced π-π stacking, involving two fused rings: pyrimidine and imidazole. Two major pairs of molecules are observed: an inverted and a direct pair, both with the glycosidic site in trans geometry (Figure 4). The first stacked pair, defined as Cg15 in Table 4, has a centroid distance of 4.003 Å, an interplanar distance of 3.265 Å, and a β angle of 35.35°, with a horizontal displacement among centroids of 2.31 Å, with a dihedral angle of 0°. The second pair, defined as Cg15–Cg16, instead exhibits a larger distance of 5.38 Å, with an interplanar distance of 3.196 Å and a higher β angle with a corresponding higher slippage of 4.328 Å (Figure 4b). The dihedral angle α is also higher, with a value of 3.99°. In the present case, the reported values are calculated from Mercury, since in PLATON, reported interactions were between pyrimidine and imidazole rings only (see Section 4 for software details).

In the previous cases, purine bases formed planar or chain-like motifs, now chains, but also describing a herring-bone pattern. In this case, weaker donor-acceptor interactions also occur, with carbon atoms acting as donors and nitrogen or oxygen as acceptors, e.g., C6-H7···O1_4, and connecting the two parallel displaced chains. Another consequence of this crystal packing is that stacking is not solely defined by parallel displaced π-π interactions, but also by T-shaped π-π stacking, which contributes to the second type of interaction between chains, quasi-perpendicular to each other and extending in different crystallographic directions. Interactions connecting quasi-perpendicular chains are also represented by C-H···N or C-H···O hydrogen bonding. An example of the T-shaped stacking is given by the protonated carbon from the imidazole ring, stacking with the imidazole aromatic ring, with a C-Cg(5-membered) distance of 3.29(18) Å and a bond angle with the plane defined by the imidazole ring of 73°. We observe that with this molecular packing, all methyl groups are now lying along the same chain/vector.

### 2.5. 1-Methyladenine

**Figure 5:**
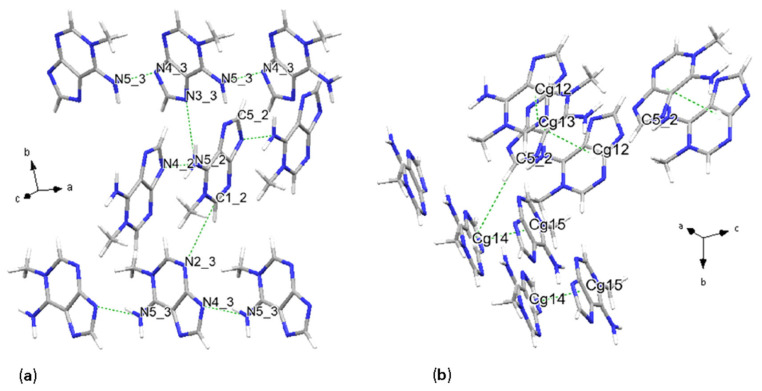
Crystal structure of 1-methyladenine. H-bonding scheme (**a**) and stacking geometry (**b**), resulting from the pairing of all four molecules defining the asymmetric unit.

Synchrotron XRPD analysis revealed that in the solid state, 1-methyladenine (1mA) molecules are organized in two chains stacking perpendicular to each other, along the crystallographic *a* and *c* directions, forming hydrogen bonds planarly within the single chain and also with the adjacent one (Figure 5). The asymmetric unit consists of four molecules, implying that each of the two stacked chains is formed by stacked pairs of asymmetric unit molecules. As in the case of 7-methylguanine, the number of molecules per asymmetric unit influences the margin of error of our findings. The overall symmetry is described by a *P*2_1_ space group [No. 4].

Parallel displaced π-π stacking occurs between pairs of molecules and is described by the centroids interactions as Cg12–Cg13 and Cg14–Cg15. For the definition of centroids, please consult Table 5. Distances among centroids are, respectively, 4.341 Å and 3.673 Å, with β angles of 36.8° and 19.37° and α angles of 14.58° and 6.37°, indicating low coplanarity among planes defined by the molecules. Interplanar distances (Cg(I)-Perp) are higher than average, with values of 3.476 Å and 3.465 Å. Rotation angles of 170(13)° and 176(19)° infer an inverse configuration of centroids; the glycosidic sites are in a trans conformation in both cases. As in the case of 7mG, the chains are connected by H-bonding and T-shaped π-π stacking. However, in the case of 1mA, the π-π interactions connecting different chains are not reported after the analysis using PLATON. The reason is that they are weaker compared to the previous case. An example is the distance of 4.588 Å between C5_2-H2_2···Cg14, compared to 3.29(18) Å in the case of C-Cg(5-membered) of 7mG. So, the predominant interactions connecting semi-perpendicular chains of molecules in the case of 1-methyladenine are of the proton-acceptor type. These interactions among different chains consist of N-H···N and C-H···N hydrogen bonds, where the amino groups (labeled N5 in this case) act as donors for N3 and N4, or N4 and N2 depending on the starting molecule of the asymmetric unit. Similarly, the C1 and C5 sites act as proton donors to N2 or N3, and the methyl group C6 to N2 or N4.

### 2.6. 3-Methyladenine Monohydrate

Solving the crystal structure of the as-purchased 3mA resulted in a monohydrate form. Pairs of molecules are forming co-planar donor-acceptor bonds, and bonding in the same plane with water molecules. These molecules stack approximately in directions of *a* − *b* and *a* + *b* with noticeable offset, forming chains. The crystal symmetry is described by a *P*2_1_/*c* [No. 14] space group.

**Figure 6:**
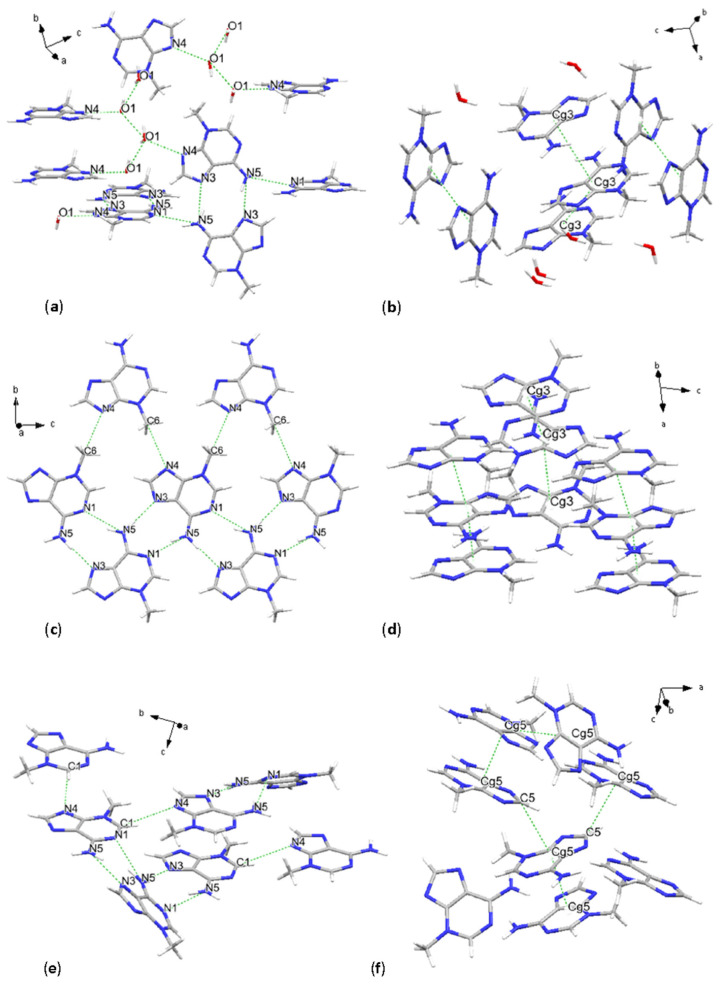
Crystal structures of 3-methyladenine monohydrate (**a**,**b**), 3-methyladenine (**c**,**d**) and 3-methyladenine polymorph (**e**,**f**). H-bonding scheme (**a**,**c**,**e**) and stacking geometries (**b**,**d**,**f**).

Donor–acceptor bonds consist of N-H···N, O-H···O between water molecules and C-H···N, where the first case consists of the amino group acting as a donor and forming two bonds with adjacent molecules, and the last case consists of weak interactions competing for the same acceptor site N1 (Figure 6a). Moreover, an O-H···N interaction forms between the water molecule O1 and N4, with O1-H donating a proton to N4. Stacking interactions are represented by parallel displaced π-π stacking between 5-, 6- and 9-ring centroids. In the case of centroids defined on purine rings, their distance is 5.010(4) Å and 4.485(4) Å according to the translation from the asymmetric unit, with a horizontal displacement of 3.638 Å and 3.072 Å, implying a high value for the β angle, respectively, 46.6° and 43.2° (Figure 6b). Interplanar distances are 3.444(3) Å and 3.268(3) Å; meanwhile, the geometry of the bases is direct, with the glycosidic site stacking in cis position, and inverted, with the glycosidic bond in trans configuration. Torsion angles among planes defined by the atoms of the centroid, with a value of 0°, indicate full coplanarity.

### 2.7. 3-Methyladenine

The in situ high temperature XRPD analysis revealed the occurrence of two major structural changes in the 3mA compound, at temperatures of approximately 100 °C and 220 °C. For this reason, the first experiment consisted of heating to 100 °C, letting the system stabilize, and cooling back to room temperature before collecting the powder pattern. The solved crystal structure revealed the complete removal of water and the synthesis of 3mA. An impurity was found during the experiment, consisting of a high-temperature polymorph, which will be discussed in the next sub-chapter. After a final Rietveld refinement, we have quantified this impurity as 4.4 wt. % of the full powder. Please refer to the Supplementary Materials (Figure S9) for the final Rietveld refinement. The crystal structure of the main constituent of the mixture consisted of the pure 3mA phase, whose crystal packing is described by planes of molecules interacting with adjacent molecule planes via donor–acceptor bonds and π-π interactions. The symmetry can be described by a space group *P*2_1_/*c* [No. 14].

Different from the case of the 3mG system, where the configuration after water removal essentially stayed planar, in this case, the packing configuration changed from chains of molecules stacking one in *a − b* and the other one in *a* + *b* directions, to planes of atoms indefinitely stacking in the crystallographic *a* direction. The hydrogen-bonding scheme preserves the similarity of N5-H3···N1 and N5-H4···N3 interactions relative to the monohydrate, whereas site N4, previously bonded to water, now shares a proton with the methyl group, in the bond C6-H6···N4 (Figure 6c and Table 6). Parallel displaced π-π stacking occurs between purine centroids (9-membered), with distances of 3.291(16) Å and 3.814(16) Å and interplanar distances Cg(I)-Perp of 3.223(12) Å and 3.640(12) Å. This finding might suggest some underlying issues in the crystallinity of the compound, in the modeling, or a reduced overall stability due to the removal of crystal water.

Rotation angles of 180° in both cases indicate an inverted configuration, with the glycosidic site in trans geometry. An α angle of 0° indicates coplanarity of the planes defined by the pair of stacked purine bases (Figure 6d).

### 2.8. 3-Methyladenine Polymorph

The high-temperature in situ synchrotron XRPD analysis revealed that after water removal at 100 °C, the 3-methyladenine system undergoes other significant crystallographic transitions starting to occur at a temperature of 167 °C according to our heating step interval, and subsequently being completed at a temperature of 220 °C, after a stabilization period of 15 min (Figure S13). The crystal structure solution of the compound cooled back to room temperature indicates an increase in symmetry, from monoclinic to tetragonal, possessing the space group P4_3_2_1_2 [No. 96]. Please note that there is an uncertainty regarding the enantiomorphic space group P4_1_2_1_2 [No. 92], which could not be distinguished based on XRPD data. The N5-H3···N1 and N5-H4···N3 interaction scheme is maintained as in the previous cases for the other 3mA, whereas site N4 is now accepting a proton from C1, forming a C-H···N hydrogen bond (Figure 6e).

Molecular packing is now not fully described by planar chains or molecules. In fact, the α angle between one of the two types of parallel-displaced π-π stacked centroids has a value of 40.5°. This means that molecules are also forming X-H···Cg interactions consisting of C5-H2···Cg5, with an X(proton donor)-Cg distance of 3.479(10) Å and a bond angle with the plane defined by the pyrimidine + imidazole ring of 37°. The Cg-Cg interaction with the same pair of molecules has a 5.442(5) Å centroid to centroid distance, a β angle of 28.7° and a rotation angle of 27(1)°. In the second pair of molecules after another translation of the asymmetric unit, the π-π stacking is nearly parallel, with a β angle of 2.4°, and a centroid to centroid distance of 3.449(4) Å, an α angle of 0.6°, and a rotation angle of 22(1)°. In both cases the glycosidic site is in a trans configuration (Figure 6f).

### 2.9. 7-Methyladenine

The solved crystal structure of 7-methyladenine (7mA) revealed two chains of molecules elongating perpendicularly to each other, similar to those observed for 1mA and 7mG. These chains stack along the crystallographic directions *b* + *c* and *b − c*. The symmetry is described by the space group *P*2_1_2_1_2_1_ [No. 19] and the asymmetric unit consists of one molecule.

**Figure 7:**
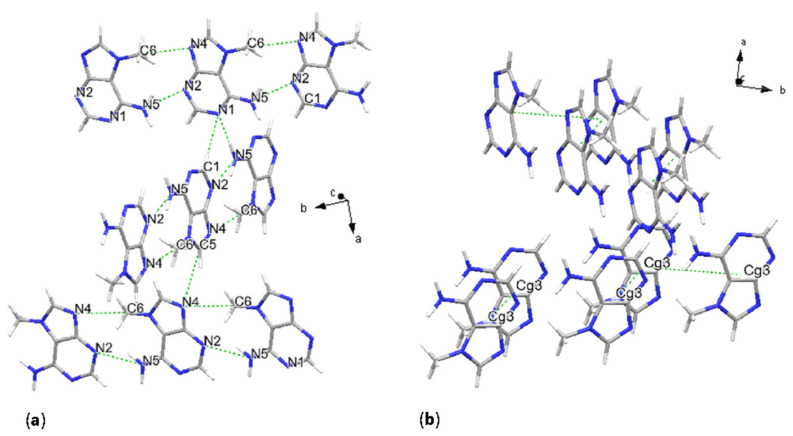
Crystal structure of 7-methyladenine. H-bonding scheme (**a**) and stacking within the unit cell (**b**).

In this case T-shaped π-π stacking is entirely missing, with perpendicular chains interacting only through H-bonds. These consist of N-H···N and C-H···N bonds. The amino group N5 shares protons both within the plane described by the first chain of molecules, interacting with the N2 site, and with the perpendicular chain through interaction with N1 (Figure 7a and Table 7). Site C5 also forms a hydrogen bond with N4 of the perpendicular chain. Within planar donor–acceptor interactions, the methyl group C6 bonds with N4. Parallel displaced π-π stacking is defined by 9-centroid distances of 5.102(7) Å and 4.636(6) Å. Interplanar distances are higher than average, as in the case of 1mA, resulting in values of 3.441(4) Å and 3.417(4) Å. The stacked centroids have a high degree of horizontal displacement, with β angles of 47.6° in both cases. Rotation angles are also consistent, with values of 0° indicating direct stacking, and the glycosidic sites indicate a cis configuration. α angles of 0° indicate no angular differences between the molecular planes (Figure 7b).

## 3. Discussion

The first objective of the present study was to determine the steric contribution of methylation in nucleobases in the solid state. Concerning *N*-methylated guanines, a study by Bald et al., 2011 [38] investigated the effect of methylation on intermolecular interactions between guanine molecules using scanning tunneling microscopy (STM) and density functional theory with empirical dispersion correction (DFT-D). This study was carried out on a graphite surface at the liquid–solid interface, with a focus on 2D motifs that were stabilized by hydrogen bonding. In the case of 1mG, molecules formed 2D windmills interacting with N9-H···N7, N2(amino)-H···N3 and N2(amino)-H···O hydrogen bonds. Instead, in the solid state, we observe ribbons with the following H-bond scheme, in IUPAC notation: N9-H···N3, N2(amino)-H···N7, and N2(amino)-H···O. Another DFT study conducted by Paragi et al., 2013 [16] predicted the plausibility of 7mG clusters with and without stabilizing anions at solid–liquid and solid–gas interfaces. These authors proposed either ring clusters due to a cooperativity effect of H-bonding within rings, or ribbons. Our findings indicate that in the solid-state, clusters of 7mG can be observed as in the latter case. Nonetheless, precise modeling of this compound was compromised by the number of parameters in the refinement. For this reason, although the cluster and donor-acceptor geometries may be accurate, a comparison with the precise distances reported by Paragi et al., 2013 [16] is not possible. Another finding from the previously mentioned study was a comparison of the clusters and H-bonding distances between their calculations and the crystal data of 7H-guanine reported by Guille and Clegg, 2006 [21], finding agreement with their calculations of the ribbon geometries for 7mG and 7H-guanine. Our findings indicate that the bonding scheme N1-H1···N3, N2-H2···N9, and N2-H2···O6 is indeed present in both 7H-guanine after Guille and Clegg, 2006 [21], and 7mG, confirming that in the solid state, this interaction geometry is maintained. However, we point out that in the solid state, whilst ribbon clusters in 7H-guanine form indefinitely extended planes that stack with parallel stacked π-π geometries, in 7mG, these ribbons form π-π stacked molecules forming chains along the *b* and *a − b* axial directions. This implies that packing is also defined by other intermolecular interactions, which consist of C-H···N or C-H···O hydrogen bonding among differently oriented chains, whereas stacking is not only defined by parallel displaced π-π interactions, but also by T-shaped π-π stacking. Concerning *N*-methylated adenines, a comparison similar to that in Paragi et al., 2013 [16] cannot be drawn for 7mA, since the crystallographic data for anhydrous adenine reported by Mahapatra et al., 2008 [22] consists of 9H-adenine. However, comparing their findings with 9mA after Kistenmacher et al., 1977 [19], we notice that in this last case, the dipole–dipole interactions describe quartets, whereas for 9H-adenine one observes a ribbon pattern. Thus, we can say that for 7mG and 7H-guanine, and 9mA and 9H-adenine, the steric contribution of the methyl group implies new interactions forming among the molecules in the crystalline state, compared to the case of pure guanine and adenine and protonated at the same N site as the methyl group. These interactions consist of T-shaped π-π stacking interactions and C-H···N donor-acceptor bonds in the case of 7mG and C(methyl)-H···N3 bonds in 9mA.

Overall, our results show that packing in methylated nucleobases is represented by planarly configured stacked molecules, forming planes with stacking infinitely in a single crystallographic direction, as in the cases of 1mG, 3mG sesquihydrate and 3mA, or planar chains stacking along different directions in the case of 3mG, 7mG and 3mA monohydrate, reaching an angle between chains close to 90° in 1mA and 7mA. In the case of the 3mA polymorph, the α angle between one of the two types of parallel-displaced π-π stacked dimers has a value of 40.5°, consisting neither of planes nor of chains. It is worth mentioning that the 3mA polymorph represents the example of the highest symmetry among the studied compounds. The increase in symmetry from low-temperature 3mA is coherent with what is generally observed also for other crystal systems at higher temperatures, for minerals and as a general tendency for crystalline materials [39,40,41]. Therefore, this polymorph might also differ when compared to trends of π-interactions.

Concerning the H-bonding motifs, besides 7mG and 1mG, ribbon configurations are also observed for 3mG sesquihydrate, 3mG and 3mA. On the other hand, 1mA, 3mA monohydrate and 7mA resulted in only two points of contact between coplanar molecules along the chains, whereas the amino group shares a proton with both the coplanar and the oriented second chain.

Other existing research has aimed at studying methylation effects on purine nucleobases regarding the interfacial structures of 1mA, 3mA, 7mA and 9mA on gold nanoparticles using surface-enhanced Raman scattering (SERS) [18]. These findings indicated that the position of the methyl group on the purine ring of adenine led to distinct surface-binding schemes. DFT calculations predicted energetic stabilities in the gas phase as, in increasing order, 1mA, 3mA, 7mA and 9mA. The overall energetic stability of a system is determined by the combined effects of all interactions within it.

A second objective of the present study was to determine π-π stacking interaction geometries and abundances among the compounds studied. The study by Hunter and Sanders 1990 [31] proposed an original model, which was validated by Wheeler, 2025 [33], indicating that in the stacking of aromatic systems, the interplay between electrostatic and van der Waals (vdW) components influences the stacking geometries. In particular, the vdW contribution dominates at lower horizontal displacements, whereas electrostatic forces would be more relevant only at higher slippages. However, this last contribution is weaker than the vdW forces on the overall interaction energy.

This is summarized by the following formula, after Hunter and Sanders 1990 [31] and Wheeler 2025 [33].

(1) $$E_{int}^{HS}=E_{elec}^{HS}+E_{vdW}^{HS}=\sum_{i\epsilon A}\sum_{i\epsilon B}\frac{q_{i}q_{j}}{R_{ij}}+\sum_{i\epsilon A}\sum_{i\epsilon B}k_{i}k_{j}\left(Ce^{-\alpha R_{ij}}-\frac{A}{R_{ij}^{6}}\right)$$

where *R* denotes the horizontal displacement at a fixed vertical separation. Notably, no vertical displacement is accounted for in the formula, since in the original model, this distance was kept fixed at a reference value, given the qualitative and conceptual original scope of the model.

The findings from Wheeler 2025 [33] and Hunter and Sanders 1990 [31], applicable both in solution and qualitatively in the solid-state, suggest that for nearly face-to-face π-stacked systems, dispersion forces (vdW) dominate. After a certain lateral displacement, depending on the system studied, at around 3 Å in the case of benzene parallel stacked dimers, this contribution to the interaction potential falls off in favor of electrostatic contributions.

These contributions also indicate that a face-to-face geometry is unfavorable unless relevant polarization effects are involved. *N*-methylated adenine and guanines are polarized owing to the methyl, oxo and amino functional groups. This is another factor to consider when assessing ideal interaction geometries and energies. In the case of nearly face-to-face geometry, following Hunter and Sanders’ predictions, the functional groups are expected to be positioned in a trans configuration. This is indeed observed in 3mA, where the 9-membered centroids have a slippage of 0.667 Å and 1.137 Å. In this case, our findings are consistent with the original model proposed by Hunter and Sunders, in which the functional groups are positioned diametrically opposite. Also, in one of the molecules in 9mG from Stolar et al. 2020 [9], one of the 9-membered centroid distances with the lowest slippage, 0.389 Å, has an inverted trans configuration. Another case in the present study is the 3mA polymorph, where the slippage of 0.147 Å of two 9-membered centroids is associated with direct stacking. Charges are expected to be balanced due to the rotation of around 22° between one another, and the trans positioning of functional groups.

**Figure 8:**
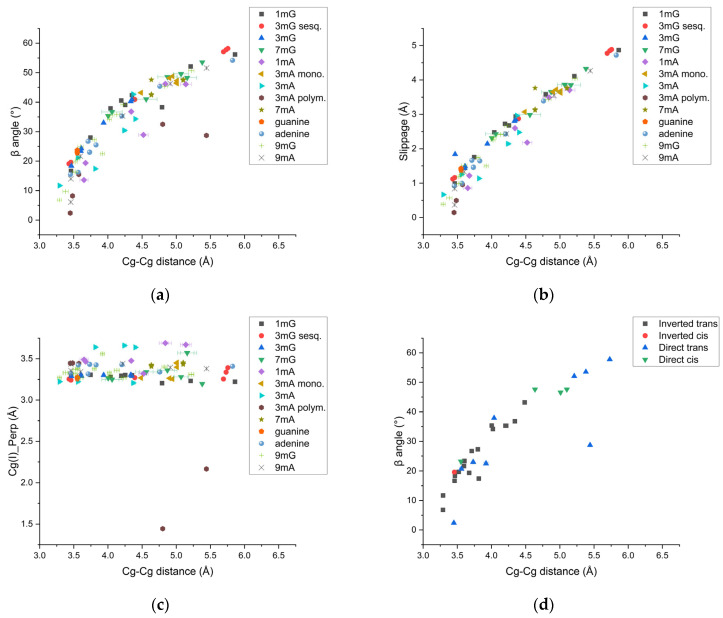
Stacking parameter trends for methylated and pure nucleobases. 5-, 6- and 9-membered centroid distances were correlated to the β angle (**a**), horizontal displacement (slippage) (**b**) and interplanar distances (Cg(I)_Perp) (**c**). The low interplanar distances in this last case were attributed to the high alpha angle for the 3mA polymorph. Please note that also parameters from guanine, adenine, 9mG and 9mA are plotted in these graphs. The plot in (**d**) relates the previous stacking parameters considering only 9-membered centroids, with inverted, direct, trans and cis geometries.

The plots in Figure 8 were obtained after combining and comparing our results for 5-, 6- and 9-membered centroids with other already solved pure and *N*-methylated nucleobases, namely 9mA [19], 9mG [9], 7H-guanine [21] and 9H-adenine [22]. All of them are characterized by a quasi-linear trend of the relationship between centroid-to-centroid distances and β angles or slippage (Figure 8a,b). These plots allow a vertical distance (*x*-axis) to be related to the horizontal displacement (*y*-axis). For centroid distances relative to the interplanar distances, we did not observe a clear trend (Figure 8c). The non-linearity in the previous two cases is here inferred to be consistent with the findings of Hunter and Sanders, 1990 [31] and Wheeler, 2025 [33]; specifically, the slight change in the slope is related to the change in contribution on the stacking interactions and geometries from vdW interactions to an electrostatic component, where at higher horizontal displacements (higher β), the interaction is dominated by the last one, which decreases linearly according to the formula. A fit of the data from all centroids from the compounds from the present study, together with 9mA, 9mG, 9H-adenine and 7H-guanine, is modeled according to the findings of Hunter and Sanders 1990 [31].

**Figure 9:**
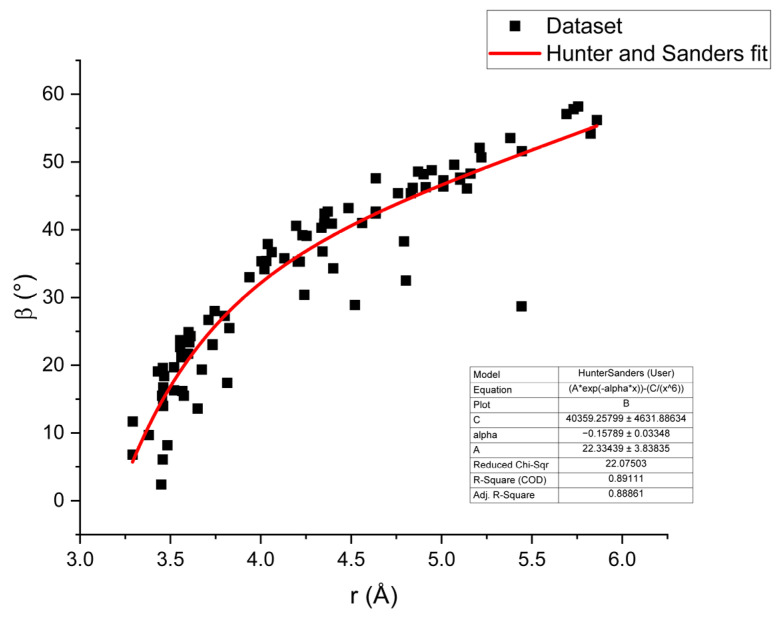
Modeling of data from all 5-, 6- and 9-centroid-to-centroid distances of corresponding compounds from the present and other studies using a modified and generalized Hunter and Sanders Equation (1).

Figure 9 shows the β angle plotted versus the Cg-Cg distance *r*. Empirically, the observed trend of the data can be fitted utilizing the generalized van der Waals interaction term $E_{vdW}=A\cdot \;e^{-\alpha \cdot r}-\frac{C}{r^{6}}$ from the Hunter–Sanders model for π-π interactions, where ***A***, *C* and *α* are standard parameters (see Hunter and Sanders, 1990 [31]) and *r* is here the Cg-Cg distance between two molecules. Please note that we assumed that the interaction term can be generalized, meaning that the summation over individual atoms i and j that interact with each other can be omitted and instead that the interaction between two molecules can be expressed by the same equation. Moreover, in order to justify the fit of this β angle versus Cg-Cg distance data, it must be further assumed that there exists a proportionality between the β angle and the van der Waals interaction term $E_{vdW}$. Under these two assumptions, the equation fits the data with reasonable agreement.

Based on existing research and our findings, we suggest that after a change in the electrostatic and vdW contributions between these stacked systems, the electrostatic contribution remains lower than the vdW contribution, leading to a change in the slope. In the case of the 3mA polymorph, one point in the plots of Figure 8 and lies outside the trend. This is due to the fact, that the planes defined by couples of molecules can reach an angle of 40°, as mentioned in Section 2. Consequently, the π-π interactions are weaker, whereas the C-H on the imidazole ring now forms an X-H··· π interaction. Other variations in trends relating to a pure vertical or horizontal component, the interplanar distance Cg(I)-Perp, and the slippage, and a vertical/horizontal component, given by the centroid-to-centroid distances, are also reported in Figure 8b,c.

A recent study by Sierański, 2020 [42], investigated the stacking interactions on purines with DFT calculations, reporting that interaction energies, among other parameters, depend on the centroid distances and the β angle. The energies have a minimum at distances of approximately 3.5 Å and an angular value between 0 and 50°, becoming more positive thereafter, consistent with the trend observed in Figure 8a. Moreover, Sierański 2020 [42] also conducted a population analysis on structures from the Cambridge Structural Database (CSD), finding that dimers in the solid state follow the same trend of abundances, with a peak at 4 Å and 30°, and 5 Å and 60° in the relation between centroid distances and β angles, which agrees with our findings.

The cis and trans notations, used here to describe the location of functional groups in stacked purine bases, were adopted from Jhunjhunwala et al. 2021 [27]. This also highlights the importance of adding all the possible interactions among rings. Furthermore, cis and trans geometries also allow for a comparison with the Hunter and Sanders 1990 model [31], indicating how protonation affects stacking geometries. Overall, the trans conformation is more frequently observed, because it is the most stable configuration according to steric repulsions accounted for in the model. In fact, where the cis configuration is observed, either the slippage is relevant (minimum represented by 1.4 Å in anhydrous guanine) or rotation angles > 0° are observed, as in the case of 3mG sesquihydrate. This can be observed in Figure 8d, where the abundances of direct or inverted configurations and cis/trans geometries are plotted as a function of 9-centroids distances and β angle. It can be observed that the direct cis conformation tends to have a larger β angle, reflecting higher horizontal displacements. This agrees with the prediction by Hunter and Sanders 1990 [31] of steric repulsion between functional groups, since the repulsion was not accounted for by the rotation angle gamma, which resulted in 0° after our findings. An exception to this is represented by 7H-guanine, with a β angle of around 20° and direct cis conformation; in this case, the rotation angle is also close to 0°.

A study by van Mourik et al., 2016 [43], describes the stacking interactions between adenine and 2-aminopurine in the gas phase using DFT. Specifically, their calculations accounted for variations in stacking distances, slide (corresponding to slippage in the present study), tilt and twist angle (referred to as our rotation angle gamma). Importantly, they have treated the rotation angle, or twist angle, as a key variable in DNA.

In an idealized DNA helix, excluding the occurrences of bends and twists in the DNA strand, the authors reported an expected twist of 36°. These authors also noticed that in El Hassan et al., 1997 [44], a comprehensive single-crystal XRD study on DNA oligomers was reported, showing a twist that varied from 10° up to 60°, slippage variations from −3 to 3 Å, and a tilt, which corresponds to the α angle in the present study, between −25 and +25°. However, findings from van Mourik et al., 2016 [43], on paired adenine/adenine, 2-aminopurine/2-aminopurine and adenine/2-aminopurine show that all configurations have a twist angle of at least 60°, which is not observed in DNA, potentially indicating that the stacking configuration in DNA is not optimal in terms of pure base/base stacking.

In Kagra et al., 2021 [14], the methylated adenine dimers tested have more stabilized stacking interactions than pure adenine dimers, but only when methylation does not add charge to the adenine system. For example, 1mA-1mA pair interactions decrease the stacking stability because the N1 site acts as a proton acceptor, reducing the charge of the system. Instead, if a methyl group is attached, we have introduced a charge to the system. However, when 1mA is paired with adenine, the stacking stability increases remarkably, even more than in adenine–adenine dimers. In the case of the methylation sites N2, N6 and N8, stacking increases compared to pure adenine pairs. As for methylguanine dimers, pure guanine has a proton in site N1 differently from adenine. Thus, methylation at the N1 site does not add any charge to the guanine system. Kagra et al., 2021 [14], have observed the same behavior as for adenine dimers: charged (7mG) dimers do not exhibit attraction. Neutrals have slightly higher stacking energies, so they are more stable. Other findings from them indicate that for all dimers (pure and methylated), the stacking energy increases with rotation angle. Perfectly direct stacking with approximately 0° of rotation is energetically unfavorable. Also, Sierański, 2020 [42], states that an optimal orientation is given by a 200 to 220° rotation, where in the mentioned study, this angle is referred to as the α angle.

The proposed chemical explanation for their findings is that, when charge modifications occur, electrostatic repulsions dominate. This is the case for 1mA dimers or 7mG dimers. In the case of methylation at other sites where the charge does not change, e.g., 1mG dimers, the stacking strength increases due to enhanced dispersion interactions originating from the methyl groups. In these cases, the charge distribution in nucleobases is taking place, considering that the nucleobase is attached to the ribose. In our results, methylation at site N7 also does not change the charge distribution.

Kagra et al., 2021 [14], also state that stacking distances of 3 Å are not optimal for stabilization, whereas closer to a distance of 4 Å, the energies are optimized. In the solid state, we observe shorter interplanar distances (stacking distances reported in the mentioned study), whereas the centroids-to-centroid distances are generally longer. This is due to the interplay between the contributing forces as attributed by the model of Hunter and Sanders. In Rutledge et al. 2008 [13], methylation increases stacking strength between the methylated purine nucleobase and an amino acid. This is in line with the findings from Kagra et al., 2021 [14], where they have found that stacking between methylated and pure nucleobases is stronger.

Overall, our findings aim to describe the frequency of stacking geometry parameters in the crystal structures of methylated purine nucleobases. The entirety of π-π stacking interactions is of a parallel displaced or T-shaped type. Moreover, trends of these parameters can be modeled successfully with a modified and generalized Hunter and Sanders equation, providing a qualitative explanation of the observed geometries. We herein prove the applicability of this model for the present aromatic compounds as a function of horizontal and vertical displacements. Our considerations on cis and trans, and direct and inverse conformations are not directly applicable to DNA, because in that case, necessarily what we find is cis and direct stacking, oriented around 30° as summarized by van Mourik et al., 2016 [43]. In RNA, the stacking interactions can be consecutive or non-consecutive depending on whether they occur on linear strands or bent strands of nucleic acid. In this latter case, it would consist of the inverse configuration (gamma angle of 180°) as compared to our study. Recently, Jhunjhunwala et al. 2021 [27] studied the frequencies of stacking interaction geometries in RNA nucleobases. Their findings indicate that among purine-purine stacked dimers, the majority of the interactions are represented by upward (=direct in the present study) cis configurations, since these occur between two consecutive stacked bases in the helical part of RNA. Other findings from the aforementioned study suggested that trans configurations were less common; however, these can be observed in loops in rRNA structures. In crystal structures of methylated purine nucleobases alone, we can observe a higher frequency of inverse and trans configurations. The findings from Kagra, 2021 [14], and from Sierański, 2020 [42], suggest that a rotation of 180°, thus inverted configurations, are expected to be energetically optimal for methylated and pure stacked adenine or guanine dimers in the first study, and for purines in general in the case of the latter.

To the best of our knowledge, the present findings represent the first experimental insights into solid-state packing motifs and supramolecular interactions of 1-, 3- and 7-methylated purine nucleobases. Information on π-π interactions is systematically evaluated here, and also compared to 9-methylated and pure counterparts, providing reasonable agreement with the Hunter and Sanders model.

## 4. Materials and Methods

### 4.1. Materials Availability and Recrystallization Experiments

The starting compounds considered in this study consist of six *N*-methylated guanines and adenines: 1-methylguanine (1mG), 3-methylguanine (3mG), 7-methylguanine (7mG), 1-methyladenine (1mA), 3-methyladenine (consisting of a monohydrate at the moment of the measurements) (3mA) and 7-methyladenine (7mA). The 1mG and 3mG crystalline powders were purchased from Santa Cruz Biotechnology, Inc. (Dallas, TX, USA) (≥95% and >98, respectively), while 7mG, 3mA and 7mA from Sigma-Aldrich Chemie GmbH (Taufkirchen, Germany) (≥98%, ≥99% and =97% purity) and 1mA from Thermo Fischer Scientific (Waltham, MA, USA) (≥ 98% purity). Briefly, 1mG and 3mG single crystals were grown in water–ethanol solutions from the purchased powders (for details, see Supplementary Materials). Part of the newly synthesized 3mG sesquihydrate single crystals were then crushed with a pestle in an agate mortar and the obtained crystalline powders, as well as ground 7mG, 1mA, 3mA monohydrate and 7mA pristine powders, were sealed in special soda-lime glass capillaries (WJM-Glas Müller GmbH, Berlin, Germany).

The morphology and crystallinity of the methylguanine samples considered were studied using Scanning Electron Microscopy (SEM) using a high-resolution field emission SEM (Nova Nano SEM 450—FEI Thermofisher, Waltham, MA, USA) [45].

Raman spectra of selected compounds were collected using a HORIBA, iHR 320 device equipped with a 633 nm HeNe laser (HORIBA, Kyoto, Japan), on powders mounted in quartz capillaries; however, intense fluorescence prevented the acquisition of interpretable data for the *N*-methylated guanines considered. More details and experimental results are reported in the Supplementary data file.

### 4.2. X-Ray Diffraction Analyses

X-ray powder diffraction patterns of 3mA monohydrate and 7mA were collected at room temperature using a laboratory diffractometer (STOE, Darmstadt, Germany; CuKα1, Multi-Mythen detector, operating in Debye Scherrer/transmission geometry, see also Section 4.3). Synchrotron X-ray powder diffraction patterns at room temperature of 7mG, 1mA and of 3mG and 3mA powders following the heating experiments have been collected in Debye Scherrer/transmission geometry at the Powder Diffraction and Total Scattering Beamline P02.1 at the Deutsches Elektronen-Synchrotron (DESY), Hamburg, Germany [46] at a wavelength of λ = 0.20739 Å and λ = 0.20709 Å (approx. 60 keV) using an unfocused and collimated X-ray beam with a size of 1 × 1 mm^2^. X-rays diffracted by the samples were detected using a Varex XRD 4343CT detector (150 × 150 µm^2^ pixel size, 2880 × 2880 pixels area) mounted orthogonal to the beam path with a sample-to-detector distance of approximately 1100 mm for 3mG and 2100 mm for 7mG, calibrated using LaB_6_ NIST SRM 660c as a reference [47]. The collected diffractograms were subsequently azimuthally integrated with the DAWN Science software (Version 2.25.0) [48] to one-dimensional powder diffraction patterns. Data sets were evaluated by the Rietveld method using TOPAS V.7 [49], which implements the Fundamental Parameters Approach [50] to describe the peak shape, while Chebyshev polynomials were used for modeling the background. The crystal structures were solved in real space by applying the simulated annealing (SA) global optimization approach, as implemented in TOPAS. During the SA runs, trial molecules were placed with random translational and rotational shifts. The random choice of the translational/rotational shifts leads to a diverse exploration of the phase space, while the choice of the target temperature T determines the probability P = exp{−(R_trial_ − R_prev_)/T} [51] in which the trial structure is accepted, where R_trial_ and R_prev_ are, respectively, the value of the figure of merit (FOM) function of the trial and the previously accepted configurations. For the final Rietveld refinement, microstructural parameters were released, and after the introduction of restraints, all atomic positions and isotropic displacement parameters were refined.

Single crystal X-ray diffraction (SC-XRD) data were collected for 1mA and 3mG sesquihydrate using a Rigaku Oxford Diffraction SuperNova (Cu Kα) four-circle diffractometer equipped with an Atlas CCD plate detector (Rigaku, Tokyo, Japan). Data collection and processing were carried out using the CrysAlisPro software [52]. Measurements were carried out with an imposed temperature of 100 K (cooled by liquid N2). Using Olex2 [53] the structure was solved with the SHELXT [54] structure solution program using Intrinsic Phasing and refined with the SHELXL [55] refinement package using Least Squares minimization. Missing secondary atom sites were located from the difference Fourier map. Non-hydrogen atoms were refined using individual, anisotropic displacement parameters. Carbon atom-bound hydrogen atoms were positioned geometrically and refined riding on their respective parent atoms. *U*_iso_(H) was fixed at 1.5 (CH_3_) or 1.2 (all other H atoms) of the parent atom’s *U*_eq_ isotropic displacement parameter. The fully refined data were reviewed using PLATON (Version 141123, 2023) [56]. Empirical absorption correction was implemented using spherical harmonics in the SCALE3 ABSPACK scaling algorithm [57].

### 4.3. Thermal Analyses

In situ high-temperature XRPD measurements were conducted on grounded powders of 3mG sesquihydrate crystals previously grown in solution. Data was collected at a laboratory diffractometer (STOE, Darmstadt, Germany; CuKα1, Multi-Mythen detector, operating in transmission geometry) equipped with a Cryostream/Cobra HT device. The experiment consisted of 5 heating steps covering a temperature range of 20–200 °C and a final measurement at room temperature. Data collection time was 5 h for each temperature step. Other thermal analyses for the 3mG system consisted of differential thermal analysis and thermogravimetric analysis (DTA-TGA) that were carried out using a STA 449 FS-Jupiter device (Netzsch, Selb, Germany). Then, 15.4 mg of compound were placed in an aluminum oxide crucible and heated from a temperature of 20 °C to 250 °C with a heating rate of 5 K/min in a 240 mL/min Ar-stream.

In situ high-temperature synchrotron XRPD was also done on 3mA. A first experiment on 3mA monohydrate consisted of heating steps of 20 °C from a starting temperature of 27 °C until 87 °C, with a heating rate of 6 °C/min and 5 min acquisition time; from this temperature, it was heated to 107 °C and stabilized for 15 more minutes, before returning to room temperature with a final data collection. These experiments revealed structural changes at a temperature of approximately 100 °C. A second experiment consisted of heating the as-purchased 3mA monohydrate between 27 and 220 °C at a heating rate of 6 °C/min and a stabilization step at the highest temperature of 45 min before cooling down to room temperature.

### 4.4. Geometrical Parameters of π-π Interactions and Site Labeling

Molecular geometry calculations were carried out by PLATON (Version 141123, 2023) [56], whereas the crystal structure graphics were generated using the software Mercury (Version 2022.2.0) [58] and the coordinate systems using the software Diamond 3.2 [59].

**Figure 10:**
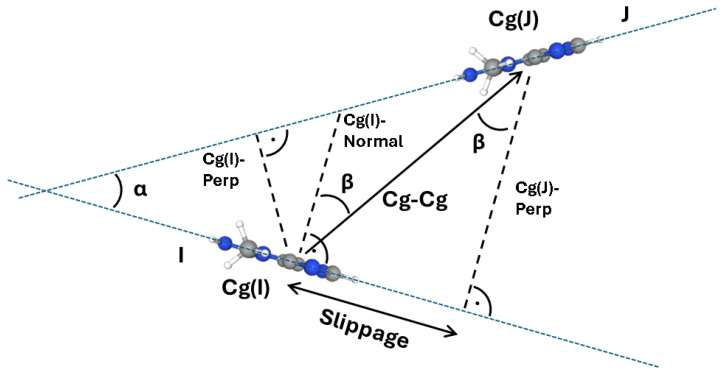
Geometrical parameters scheme for describing stacking interactions. In this example the molecule 3-methylguanine was exemplarily used.

Concerning the parameters considered in this study for describing stacking interactions, they will be defined as follows: centroids were defined for pyrimidine (6-membered), imidazole (5-membered) and purine (9-membered) rings as the geometrical center of the rings. Particular attention would be paid to the 9-membered centroids. When interacting, the two centroids involved in the stacking interaction and originating in planes I and J, would be called Cg(I) and Cg(J). Cg(I)-Perp is defined as the perpendicular distance of Cg(I) on the plane J; it can be understood as the interplanar distance. The dihedral angle α is defined here as the angle between two intersecting planes, where a plane is defined by the atoms of a respective molecule. β is defined as the angle defined by the vector originating from Cg(I) towards Cg(J) and the normal Cg(I)-Normal originating from Cg(I) to the plane J, or likewise by the perpendicular distance Cg(J) on plane I (Cg(J)-Perp) and can be calculated by arccos(*a*/*i*) where *a* is Cg(J)-Perp and *i* is the Cg-Cg distance, and it represents the angle describing how displaced the stacked molecules are, or in other words, the entity of the parallel displaced stacking. A related parameter is the slippage, equivalent to the horizontal displacement, calculated as the distance between Cg(I) and the perpendicular projection of Cg(J) on ring I (Figure 10).

Here we will also refer to inverted or direct configurations when the pyrimidine or imidazole rings of the stacking purine nucleobases would be paired with the opposite one, e.g., pyrimidine stacking with imidazole and vice versa (inverted) or pyrimidine stacking with the pyrimidine ring (direct). In this way, rotational angles γ have been defined as the torsion between C-O(oxo)mol.1-C-O(oxo)mol.2 for guanines and C-N(amino)mol.1-C-N(amino)mol.2. Values from 0° to 90° indicate a direct pairing and values from 90° to 180° an inverted pairing.

Another aspect that is required to be considered is whether the site involved in the glycosidic bond (N9 according to IUPAC nomenclature) would be on the same or opposite side of the molecules, referred here as cis or trans configurations, following the nomenclature proposed by Jhunjhunwala et al., 2021 [27]. Nomenclature for each molecular site is arbitrary in this study, given the naming scheme from the starting model. Please adhere to nomenclature when applying the findings of this study to broader chemical contexts. For this reason, during the discussion we would indicate the corresponding molecular site without the IUPAC numbering scheme. For more analytical results of the H-bonds and stacking geometrical parameters, we suggest referring to the Supplementary Materials. Furthermore, when unavailable in PLATON, molecular geometry calculations for 9-membered centroids were performed individually using Mercury. In these cases, no standard deviations were available.

## Figures and Tables

**Table 1:** Crystallographic parameters of N-methylated purines solved in this study.

	1-Methylguanine	3-Methylguanine Sesquihydrate	3-Methylguanine	7-Methylguanine	
Chemical formula (sum)	*C*_6_ *H*_7_ *N*_5_ *O*	*C*_12_ *H*_20_ *N*_10_ *O*_5_	*C*_6_ *H*_7_ *N*_5_ *O*	*C*_6_ *H*_7_ *N*_5_ *O*	
Crystal system	Monoclinic	Monoclinic	Monoclinic	Triclinic	
Space group	*P*2_1_/*c*	*P*2_1_/*c*	*P*2_1_/*c*	*P* $\bar{1}$	
*a* (Å)	9.7617(1)	10.1168(1)	6.6496(2)	9.884(1)	
*b* (Å)	14.6634(2)	10.5253(1)	10.5339(9)	10.0266(4)	
*c* (Å)	9.5542(2)	15.9583(2)	12.276(1)	17.364(2)	
*α* (Å)	90	90	90	89.94(1)	
*β* (Å)	95.446(2)	91.701(1)	123.318(4)	83.599(9)	
*γ* (Å)	90	90	90	120.52(1)	
Cell volume (Å^3^)	1361.42(4)	1698.53(3)	718.6(1)	1385.2(3)	
Formula units Z	8	4	4	8	
Crystal density (g cm^−3^)	1.612	1.503	1.5266(2)	1.5838(3)	
Radiation wavelength (Å)	1.54184 (Cu/K*α*)	1.54184 (Cu/K*α*)	0.20739	0.20709	
R_p_ (%)			0.355	0.497	
R_wp_ (%)			0.576	0.724	
R1	0.0535	0.0314			
wR2	0.1583	0.0879			
GOF	1.070	1.030	1.436	1.327	
Starting angle (°2θ)	4.507	4.169	1	0.5	
Final angle (°2θ)	76.098	75.827	10.5	7.5	
F(000)	688	808			
CCDC deposition number	2442806	2442805	2423639	2442843	
	1-methyladenine	3-methyladenine monohydrate	3-methyladenine	3-methyladenine polymorph	7-methyladenine
Chemical formula (sum)	*C*_6_ *H*_7_ *N*_5_	*C*_6_ *H*_9_ *N*_5_ *O*	*C*_6_ *H*_7_ *N*_5_	*C*_6_ *H*_7_ *N*_5_	*C*_6_ *H*_7_ *N*_5_
Crystal system	Monoclinic	Monoclinic	Monoclinic	Tetragonal	Orthorhombic
Space group	*P*2_1_	*P*2_1_/*c*	*P*2_1_/*c*	*P*4_3_2_1_2	*P*2_1_2_1_2_1_
*a* (Å)	7.4193(4)	11.3072(3)	6.9779(3)	7.53319(18)	27.173(2)
*b* (Å)	24.4950(15)	5.00954(9)	12.3509(10)	7.53319(18)	5.1021(2)
*c* (Å)	7.4126(4)	13.9918(3)	8.3443(6)	23.9923(4)	4.6352(2)
*α* (Å)	90	90	90	90	90
*β* (Å)	80.734(5)	89.6737(16)	72.534(7)	90	90
*γ* (Å)	90	90	90	90	90
Cell volume (Å^3^)	1329.56(13)	792.54(3)	685.99(9)	1362.54(7)	642.62(7)
Formula units Z	8	4	4	8	4
Crystal density (g cm^−3^)	1.49027	1.40102	1.44420	1.45527	1.54166
Radiation wavelength (Å)	0.20734	1.540596 (Cu/K*α*1)	0.207344	0.207344	1.540596 (Cu/K*α*1)
R_p_ (%)	0.586	3.145	0.520	0.642	6.137
R_wp_ (%)	0.958	4.234	0.892	1.126	8.855
R1					
wR2					
GOF	1.646	10.426	1.721	1.927	6.913
Starting angle (°2θ)	0.4	4	0.6	0.8	4
Final angle (°2θ)	7.5	84	6	8	87.5
F(000)					
CCDC deposition number	2472478	2472479	2475901	2475887	2472513

**Table 2:** Selected hydrogen-bond geometry and geometrical parameters of π-π interactions of 1-methylguanine (Å, °) *.

D-H···A	d(D-H)	d(H···A)	d(D···A)	∠(D-H···A)	Symmetry Code	
N2-H2···N8	0.88	1.98	2.844(2)	167	1 − x, −1/2 + y, 1/2 − z	
N5-H5A···O2	0.88	2.11	2.893(2)	148	x, y, z	
N5-H5B···N6	0.88	2.13	2.822(2)	135	x, y, z	
N7-H7···N3	0.88	1.98	2.843(2)	168	1 − x, 1/2 + y, 3/2 − z	
C9-H9···O2	0.95	2.39	3.257(2)	152	1 − x, 1/2 + y, 3/2 − z	
Centroids interaction **	Cg···Cg distance	Cg(I)-Perp	β-angle	Slippage	Rotation angle and geometry	Symmetry code
Cg3···Cg3	3.4595(9)	3.3091(6)	16.7	0.993	180.0(1), trans	2 − x, 1 − y, 1 − z
Cg3···Cg7	4.0379(8)	3.2801(6)	37.9	2.481	82.6(2), trans	1 − x, 1 − y, 1 − z
Cg7···Cg7	5.2103(8)	3.2316(6)	52.1	4.11	79.8(3), trans	x, 3/2 − y, −1/2 + z

* More details can be found in Tables S1 and S2 in the Supplementary Materials. ** Centroids defined for pyrimidine + imidazole rings: Cg3 = N1C2C1N4C5N3C4N2C3; Cg7 = N6C8C7N9C11N8C10N7C9.

**Table 3:** Selected hydrogen-bond geometry and geometrical parameters of π-π interactions of 3-methylguanines (Å, °) *.

D-H···A	d(D-H)	d(H···A)	d(D···A)	∠(D-H···A)	Symmetry Code	
3-methylguanine sesquihydrate						
O3-H3A···N8	0.883(17)	1.887(17)	2.7680(11)	174.7(16)	x, y, z	
O3-H3B···O2	0.883(16)	1.863(16)	2.7406(10)	172.1(15)	−1 + x, y, z	
O5-H5D···O3	0.897(16)	1.875(16)	2.7694(11)	175.8(17)	x, 1/2 − y, 1/2 + z	
N9-H9···N1	0.944(16)	1.833(16)	2.7763(12)	178.6(16)	2 − x, 1 − y, 1 − z	
N10-H10A···O5	0.903(15)	2.061(15)	2.9342(12)	162.5(15)	1 − x, −y, 1 − z	
N10-H10B···O2	0.900(15)	2.012(15)	2.8798(12)	161.6(13)	2 − x, −y, 1 − z	
3-methylguanine						
N2-H1···O1	0.90(2)	1.99(2)	2.867(16)	164(2)	1 − x, 1/2 + y, 1/2 − z	
N2-H2···N5	0.90(2)	3.30(2)	4.18(2)	166(2)		
N4-H3···N1	0.946(18)	1.832(18)	2.751(17)	163.0(18)	1 − x, −1/2 + y, 1/2 − z	
Centroids interaction **	Cg···Cg distance	Cg(I)-Perp	β-angle	Slippage	Rotation angle and geometry	Symmetry code
3-methylguanine sesquihydrate						
Cg3···Cg6	3.4578(4)	3.2409(3)	19.6	1.16	159.85(8), cis	x, y, z
	5.7300(4)	3.3377(3)	57.8	4.851	10.6(2), trans	x, 1/2 − y, 1/2 + z
3-methylguanine						
Cg3···Cg3	3.604(11)	3.306(8)	23.4	1.434	180(3), trans	1 − x, − y, 1 − z
	3.465(11)	3.288(8)	18.4	1.094	180(2), trans	2 − x, − y, 1 − z

* More details can be found in Tables S1 and S2 in the Supplementary Materials. ** Centroids defined for pyrimidine + imidazole rings: 3mg sesquihydrate: Cg3 = N6C1C3N9C5N8C3N7C2; Cg6 = N1C7C10N4C11N3C9N2C8; 3mg: Cg3 = N1C1N3C2N5C5N4C3C4.

**Table 4:** Selected hydrogen-bond geometry and geometrical parameters of π-π interactions of 7-methylguanine (Å, °) *.

D-H···A	d(D-H)	d(H···A)	d(D···A)	∠(D-H···A)	Symmetry Code	
N2_2-H1_2···N5_3	0.9(3)	2.4(3)	3.36(18)	174(1)	−1 − x, −y, −1 − z	
N2_2-H2_2···O1_3	0.9(3)	1.9(2)	2.8(2)	167(1)	−1 − x, −y, −1 − z	
N1_3-H3_3···N3_2	1.0(3)	2.1(3)	3.0(3)	161(17)	−1 − x, −y, −1 − z	
C6_4-H6_4···N5	0.9(2)	2.5(2)	3.2(2)	141(17)	x, y, −1 + z	
C6-H7···O1_4	1.0(2)	2.5(2)	3.3(2)	135(18)	x, y, 1 + z	
Centroids interaction **	Cg···Cg distance	Cg(I)-Perp	β-angle	Slippage	Rotation angle and geometry	Symmetry code
Cg15···Cg16	5.38	3.196	53.55	4.328	35(21), trans	2 − x, 1 − y, 1 − z
Cg15···Cg15	4.003	3.265	35.35	2.31	180(42), trans	−2 − x, 2 − y, −1 − z

* More details can be found in Tables S1 and S2 in the Supplementary Materials. ** Centroids defined for pyrimidine + imidazole rings: Cg15 = C2_3N5_3C5_3N4_3C3_3N3_3C2_3C4_3N1_3C1_3; Cg16 = C2_2N5_2C5_2N4_2C3_2N3_2C2_2C4_2N1_2C1_2.

**Table 5:** Selected hydrogen-bond geometry and geometrical parameters of π-π interactions of 1-methyladenine (Å, °) *.

D-H···A	d(D-H)	d(H···A)	d(D···A)	∠(D-H···A)	Symmetry Code	
N5-H3···N3_4	1.01(16)	2.29(17)	3.26(16)	161(11)	−x, −1/2 + y, −z	
N5-H4···N4	1.04(13)	1.90(14)	2.83(13)	147(10)		
N5_3-H3_3···N2	1.03(14)	2.18(17)	3.14(17)	154(9)	x, −1 + y, 1 + z	
N5_3-H4_3···N4_3	1.03(17)	1.92(16)	2.91(16)	161(10)		
C1-H1···N2_4	1.01(18)	2.44(19)	3.27(18)	140(15)	1 + x, y, −1 + z	
C5_H2···N3_4	1.0(2)	2.50(18)	3.45(18)	156(14)	−x, −1/2 + y, −z	
C6_H7···N2_4	1.01(17)	2.59(16)	3.54(17)	157(10)	1 + x, y, z	
C1_3-H1_3···N3	1.0(2)	2.11(19)	3.13(19)	178(1)	1 − x, −1/2 + y, −z	
C5_4-H2_4···N2_2	1.0(2)	2.46(19)	3.4(2)	151(12)	−1 − x, 1/2 + y, −z	
C6_4-H7_4···N4_3	1.02(13)	2.50(13)	3.33(13)	140(11)	−1 + x, 1/2 + y, −z	
Centroids interaction **	Cg···Cg distance	Cg(I)-Perp	β-angle	Slippage	Rotation angle and geometry	Symmetry code
Cg12···Cg13 ***	4.341	3.476	36.8	2.6	170(13), trans	1 + x, y, z
Cg14···Cg15 ***	3.673	3.465	19.37	1.219	176(19), trans	−1 + x, 1 + y, z

* More details can be found in Tables S1 and S2 in the Supplementary Materials. ** Centroids defined for pyrimidine + imidazole rings: Cg12 = N3C3C2N4C5N1C1N2C4; Cg13 = N3_2C3_2C2_2N4_2C5_2N1_2C1_2N2_2C4_2; Cg14 = N3_3C3_3C2_3N4_3C5_3N1_3C1_3N2_3C4_3; Cg15 = N3_4C3_4C2_4N4_4C5_4N1_4C1_4N2_4C4_4. *** Centroid selected and values calculated from Mercury.

**Table 6:** Selected hydrogen-bond geometry and geometrical parameters of π-π interactions of 3-methyladenines (Å, °) *.

D-H···A	d(D-H)	d(H···A)	d(D···A)	∠(D-H···A)	Symmetry Code	
3-methyladenine monohydrate						
N5-H3···N3	1.009(7)	1.932(7)	2.883(8)	156.1(6)	−1 − x, 2 − y, −z	
N5-H4···N1	1.030(8)	2.162(9)	3.102(9)	150.7(4)	−1 − x, 1/2 + y, −1/2 − z	
O1_a-H8···O1	0.950(6)	2.452(5)	2.908(6)	109.4(3)	1 − x, −1/2 + y, 1/2 − z	
O1-H9···O1	0.950(6)	2.566(6)	2.908(6)	101.5(4)	2 − x, −1/2 + y, 1/2 − z	
O1-H9···N4	0.949(6)	2.932(8)	2.818(8)	73.7(4)		
C5-H2···N1	1.019(12)	2.550(11)	3.473(11)	150.5(8)	x, 3/2 − y, 1/2 − z	
3-methyladenine						
N5-H3···N1	1.01(2)	2.14(4)	3.02(4)	144(2)	x, −3/2 − y, 1/2 + z	
N5-H4···N3	1.03(2)	2.00(3)	3.03(3)	173(2)	x, −3/2 − y, −1/2 + z	
C6-H6···N4	1.01(3)	2.68(4)	3.25(4)	116(2)		
3-methyladenine polymorph						
N5_b-H3···N1	1.010(10)	2.053(11)	2.990(11)	153.3(7)	−1/2 − x, −1/2 + y, 7/4 − z	
N5-H4···N3	1.030(11)	2.003(11)	2.968(12)	155.0(6)	−3/2 − x, 1/2 + y, 7/4 − z	
C1-H1···N4	1.020(13)	2.577(11)	3.551(12)	159.7(8)	−1 + y, 1 + x, 2 − z	
Centroids interaction **	Cg···Cg distance	Cg(I)-Perp	β-angle	Slippage	Rotation angle and geometry	Symmetry code
3-methyladenine monohydrate						
Cg3···Cg3	5.010(4)	3.444(3)	46.6	3.638	0.0(7), cis	x, 1 + y, z
	4.485(4)	3.268(3)	43.2	3.072	180.0(9), trans	−1 − x, 1 − y, −z
3-methyladenine						
Cg3···Cg3	3.291(16)	3.223(12)	11.7	0.667	180(4), trans	1 − x, −1 − y, −2 − z
	3.814(16)	3.640(12)	17.4	1.137	180(5), trans	2 − x, −1 − y, −2 − z
3-methyladenine polymorph						
Cg5···Cg5	3.449(4)	3.446(3)	2.4	0.147	22(1), trans	−2 + y, 2 + x, 2 − z
	5.442(5)	2.167(3)	28.7		27(1), trans	−3/2 − x, −1/2 + y, 7/4 − z

* More details can be found in Tables S1 and S2 in the Supplementary Materials. ** Centroids defined for pyrimidine + imidazole rings: 3ma monohydrate: Cg3 = N1C1N2C2N4C5N3C3C4; 3ma: Cg3 = N1C1N2C2N4C5N3C3C4; 3ma polymorph: Cg5 = N2C1N2C4C3N3C5N4C2.

**Table 7:** Selected hydrogen-bond geometry and geometrical parameters of π-π interactions of 7-methyladenine (Å, °) *.

D-H···A	d(D-H)	d(H···A)	d(D···A)	∠(D-H···A)	Symmetry Code	
N5-H3···N2	1.011(17)	2.087(16)	2.949(16)	141.8(13)	x, −1 + y, −1 + z	
N5-H4···N1	1.031(19)	2.083(19)	3.112(19)	176.8(11)	1/2 − x, −1 − y, −1/2 + z	
C1_a-H1···N1	1.02(2)	2.49(2)	3.50(2)	176.6(15)	1/2 − x, −1 − y, 1/2 + z	
C5-H2···N4	1.02(3)	2.43(2)	3.37(2)	152.7(15)	−x, −1/2 + y, 3/2 − z	
C6-H7···N4	1.013(19)	2.568(16)	3.388(17)	137.7(11)	x, y, −1 + z	
Centroids interaction **	Cg···Cg distance	Cg(I)-Perp	β-angle	Slippage	Rotation angle and geometry	Symmetry code
Cg3···Cg3	5.102(7)	3.441(4)	47.6	3.766	0(1), cis	x, −1 − y, z
	4.636(6)	3.417(4)	47.6	3.766	0(2), cis	x, y, −1 + z

* More details can be found in Tables S1 and S2 in the Supplementary Materials. ** Centroids defined for pyrimidine + imidazole rings: Cg3 = N1C1N2C2N4C5N3C3C4.

## Data Availability

Crystallographic data for the structures reported in this article have been deposited at the Cambridge Crystallographic Data Centre under the following deposition numbers: CCDC 2442806, CCDC 2442805, CCDC 2423639, CCDC 2442843, CCDC 2472478, CCDC 2472479, CCDC 2475901, CCDC 2475887, CCDC 2472513. Additional data generated and analyzed during the current study are available in the Supplementary Materials, and from the corresponding author on request.

## Supplementary Materials

The supporting information can be downloaded at: https://www.mdpi.com/article/10.3390/molecules31081326/s1.
