# Supplementary material for: N-Methylated Nucleobases Crystal Structures and π-π Stacking Interactions
Source: Molecules. 2026 Apr 17;31(8):1326. doi: 10.3390/molecules31081326 (PMC13119363; doi:10.3390/molecules31081326)
Supplement: Supplementary file 1 [file molecules-31-01326-s001.zip › molecules-4223904-supplementary.pdf]

Supplementary materials

N-methylated Nucleobases Crystal Structures and π-π Stacking Interactions

Riccardo Cameli Manzo[1,2], Volodymyr Baran[1], Artem Schevchenko[2], Anastasia Sleptsova[2], Frank Hoffmann[3], Tomislav Stolar[4], Robert E. Dinnebier[2], Martin Etter[1]*

[1]Deutsches Elektronen-Synchrotron DESY, Notkestrasse 85, 22607 Hamburg, Germany. [2]Max Planck Institute for Solid State Research, Heisenbergstrasse 1, 70569 Stuttgart, Germany. [3]Universität Hamburg, Warburgstraße 26, 20354 Hamburg, Germany. [4]BAM Federal Institute for Materials Research and Testing, Richard-Willstätter-Strasse 11, 12489 Berlin, Germany.

1. Re-crystallization experiments

3-methylguanine (3mG)

10.2 mg of 3mG was dissolved in a 1:1 mixture of 2.5 ml deionized water and 2.5 ml ethanol in a 25 ml glass beaker by manual stirring. Since the powder did not dissolve very well, the beaker glass was transferred to an ultrasonic bath for 2 min, to achieve a better dissolution. After the ultra-sonic bath treatment, the powder was heated to 75°C while further manual stirring was applied.

After stopping the stirring, small particles were immediately visible on the bottom of the beaker glass. The beaker glass was sealed with Parafilm and the Parafilm was perforated with a few holes, to let the solution evaporate from the beaker glass.

After a few weeks, the solution had completely evaporated and small crystals could be found on the bottom of the beaker glass.

Putative well-grown single crystals were then separated from other crystals under an optical microscope and subjected to single-crystal X-ray diffraction measurements.

Measurements confirmed that the grown crystals are the sesquihydrate of 3mG.

1-methylguanine (1mG)

13.6 mg of 1mG was dissolved in a 1:1 mixture of 2.5 ml deionized water and 2.5 ml ethanol in a 25 ml glass beaker by manual stirring. Since the powder did not dissolve very well, the beaker glass was transferred to an ultrasonic bath for 2 min, to achieve a better dissolution. After the ultra-sonic bath treatment, the powder was heated to 70 °C while further manual stirring was applied.

Stirring was stopped and the beaker glass was sealed with Parafilm and the Parafilm was perforated with a few holes, to let the solution evaporate from the beaker glass.

After a few weeks, the solution had completely evaporated and small crystals could be found on the bottom of the beaker glass.

Putative well-grown single crystals were then separated from other crystals under an optical microscope (see also figure S1) and subjected to single crystal X-ray diffraction measurements.

Measurements confirmed that the grown crystals are the anhydrate of 1mG.

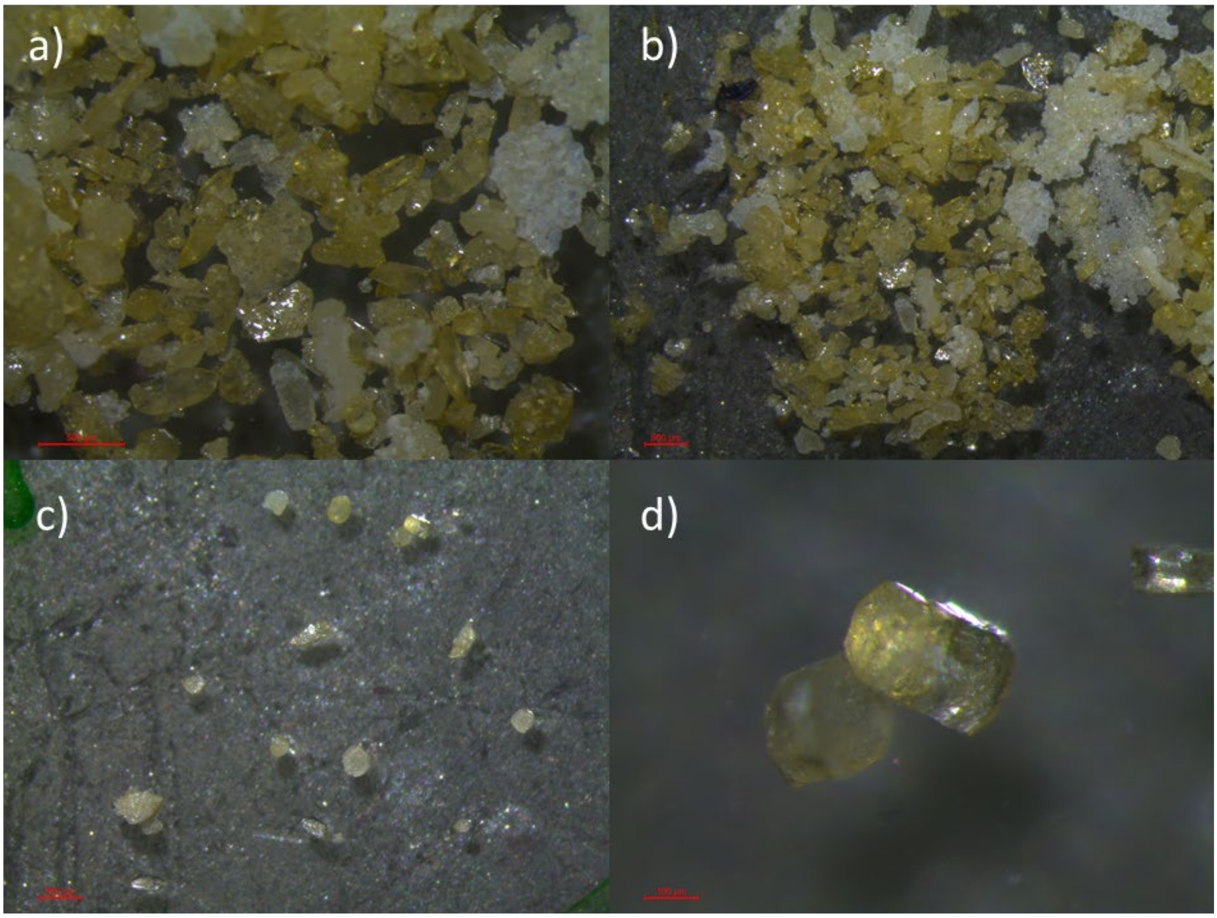

Figure S1: a)-d) Optical microscope photographs with different zoom magnification of 1mG crystals (single up to multiple twinned). Scale bars are respectively 500 µm (a), 500 µm (b), 500 µm (c) and 100 µm (d).

2. SEM images

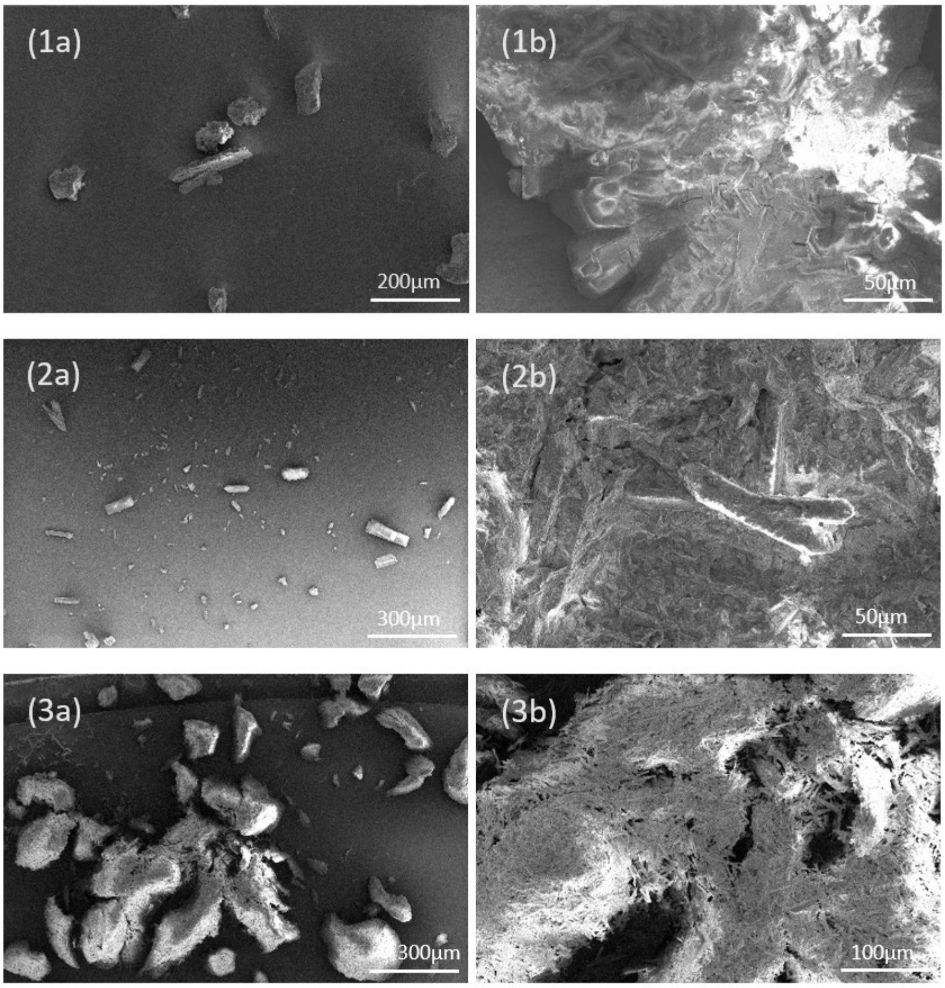

Figure S2. SEM images of 1) 1mG, 2) 3mG sesquihydrate and 3) 7mG at different scales.

3. Diffraction patterns of methylated nucleobases and corresponding Rietveld refinements

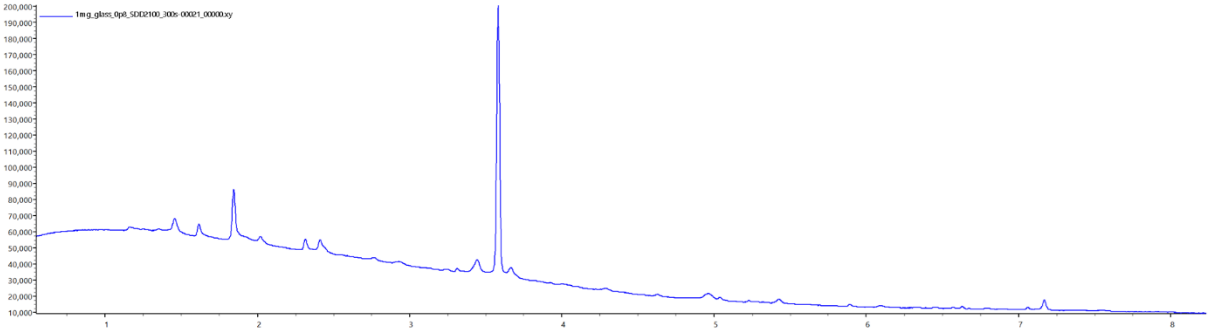

Figure S3. 1-methylguanine diffraction pattern of powder as purchased. The x-axis is given as 2θ in °, while the y-axis represents the intensity in arbitrary units. Re-crystallization experiments were carried out as described above. Radiation wavelength: 0.20709 Å.

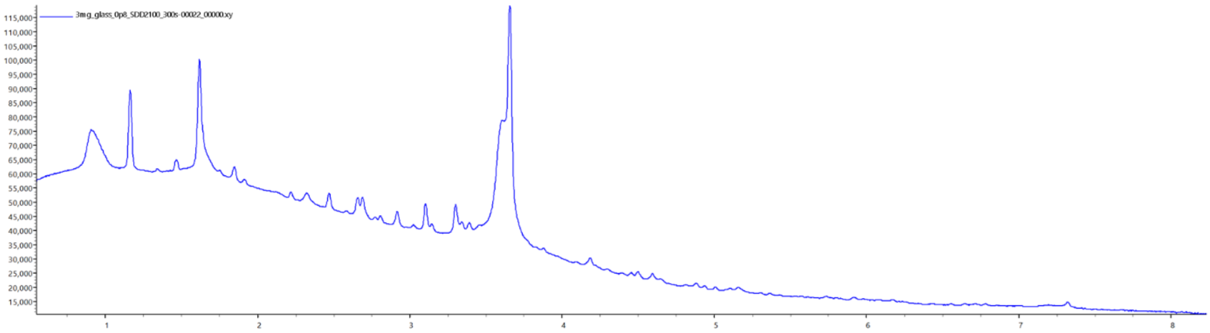

Figure S4. 3-methylguanine sesquihydrate diffraction pattern as purchased. The x-axis is given as 2θ in °, while the y-axis represents the intensity in arbitrary units. Re-crystallization experiments were carried out as described above. Radiation wavelength: 0.20709 Å.

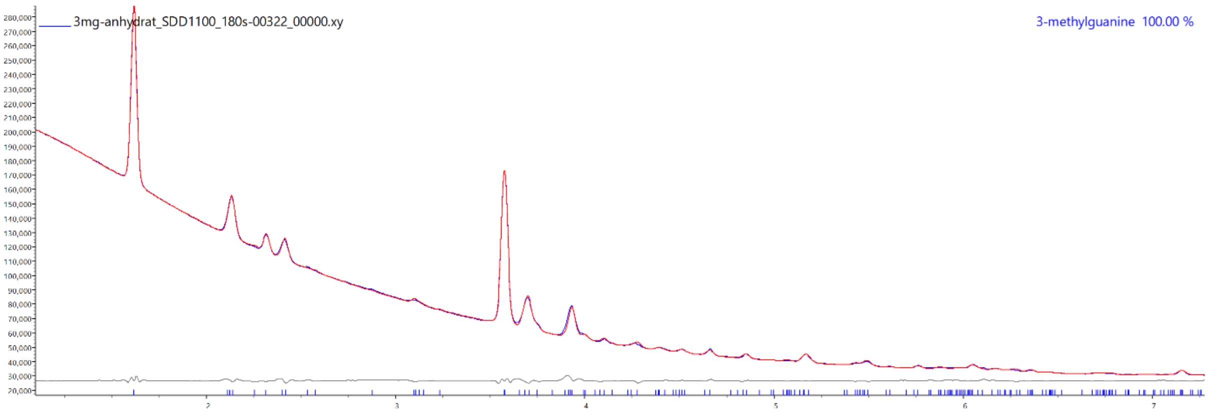

Figure S5. 3-methylguanine Rietveld refinement. The x-axis is given as 2θ in °, while the y-axis represents the intensity in arbitrary units. Radiation wavelength: 0.20739 Å.

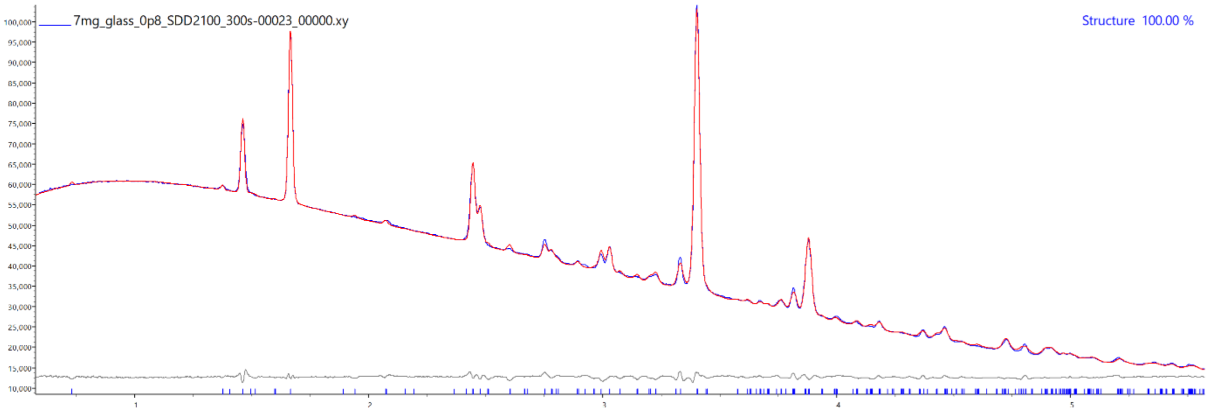

Figure S6. 7-methylguanine Rietveld refinement. The x-axis is given as 2θ in °, while the y-axis represents the intensity in arbitrary units. Radiation wavelength: 0.20709 Å.

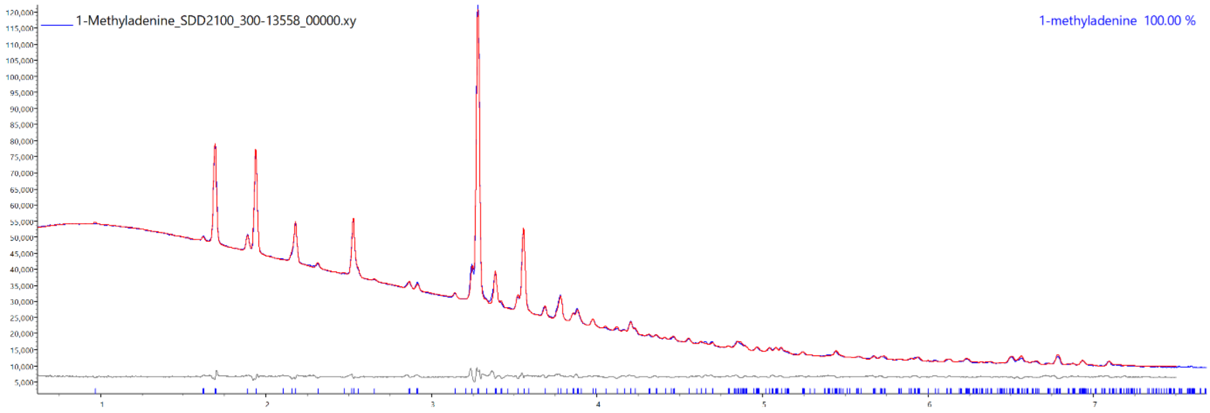

Figure S7. 1-methyladenine Rietveld refinement. The x-axis is given as 2θ in °, while the y-axis represents the intensity in arbitrary units. Radiation wavelength: 0.20734 Å

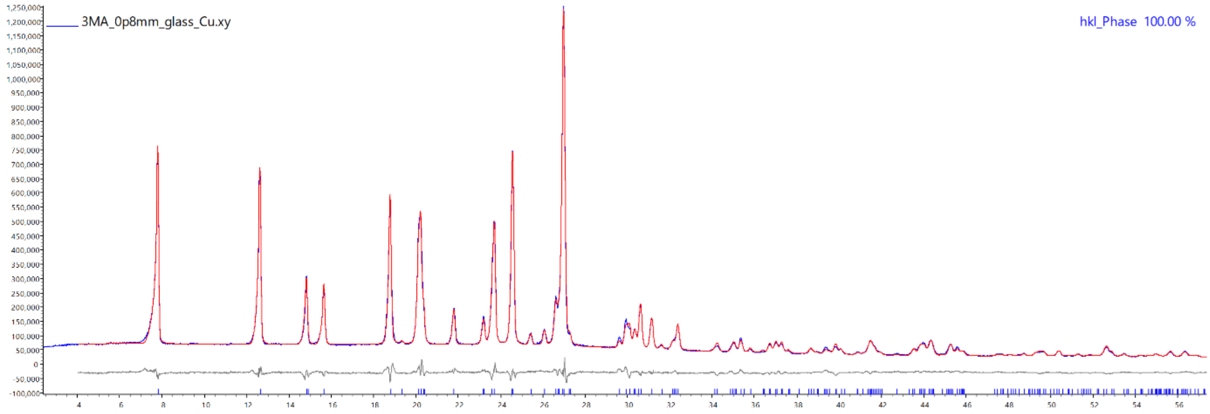

Figure S8. 3-methyladenine monohydrate Rietveld refinement. The x-axis is given as 2θ in °, while the y-axis represents the intensity in arbitrary units. Radiation wavelength: 1.540596 Å (Cu/Kα1).

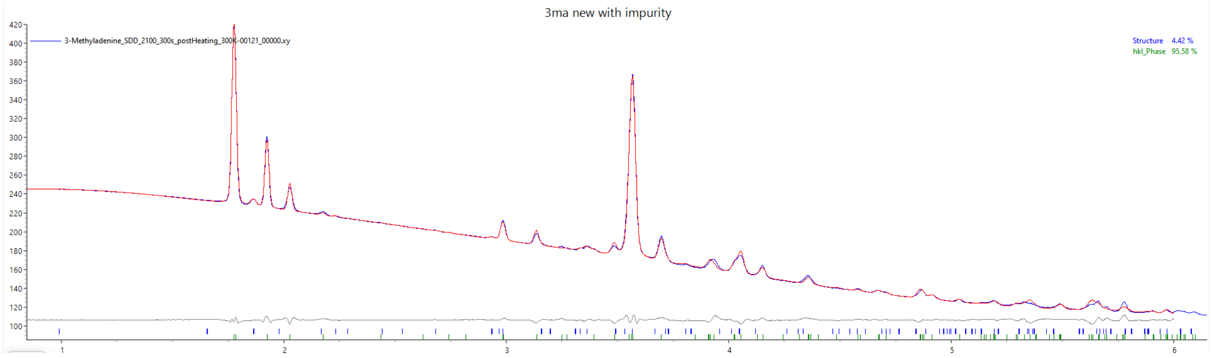

Figure S9. 3-methyladenine measured at RT after heating until 380K. Calculated diffraction pattern modeled with high-T polymorph as an impurity (wt. ca. 4%). The x-axis is given as 2θ in °, while the y-axis represents the intensity in arbitrary units. Wavelength: 0.207344 Å.

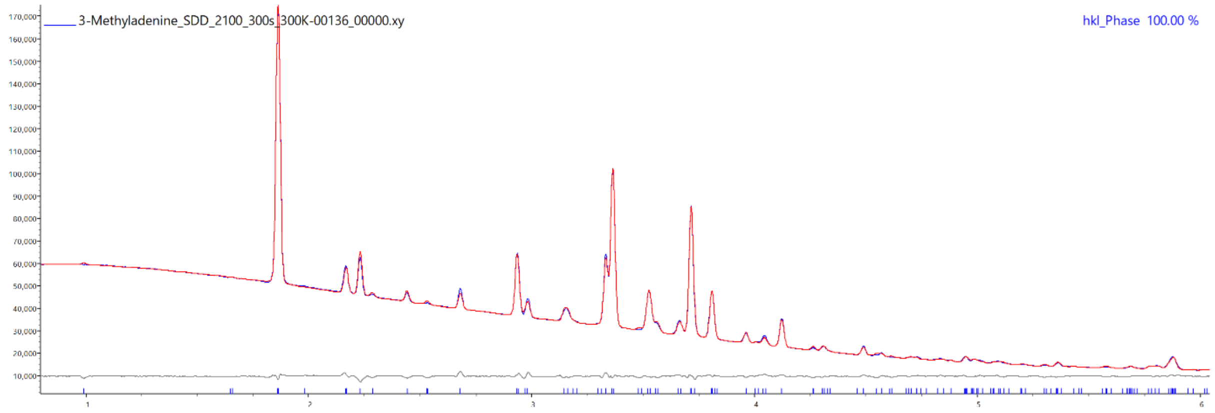

Figure S10. 3-methyladenine polymorph forming at ca. 200 °C, calc. diffraction pattern modeled with an orthorhombic unit cell. Space group $P2_12_12_1$. The x-axis is given as 2θ in °, while the y-axis represents the intensity in arbitrary units. Radiation wavelength: 0.207344 Å.

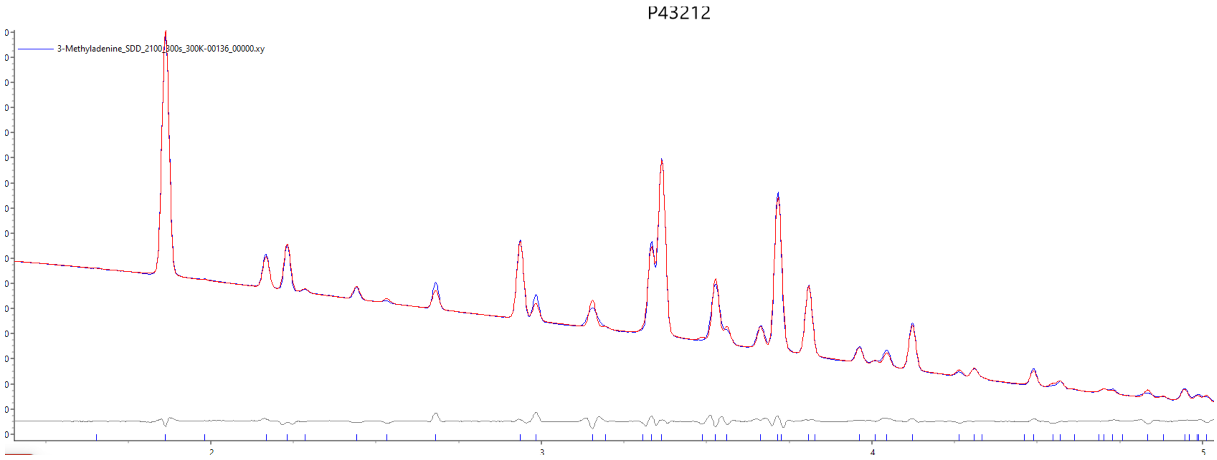

Figure S11. 3-methyladenine polymorph as prev. case, calc. pattern modeled with a tetragonal unit cell. Space group $P4_32_12$. The x-axis is given as 2θ in °, while the y-axis represents the intensity in arbitrary units. Radiation wavelength: 0.207344 Å.

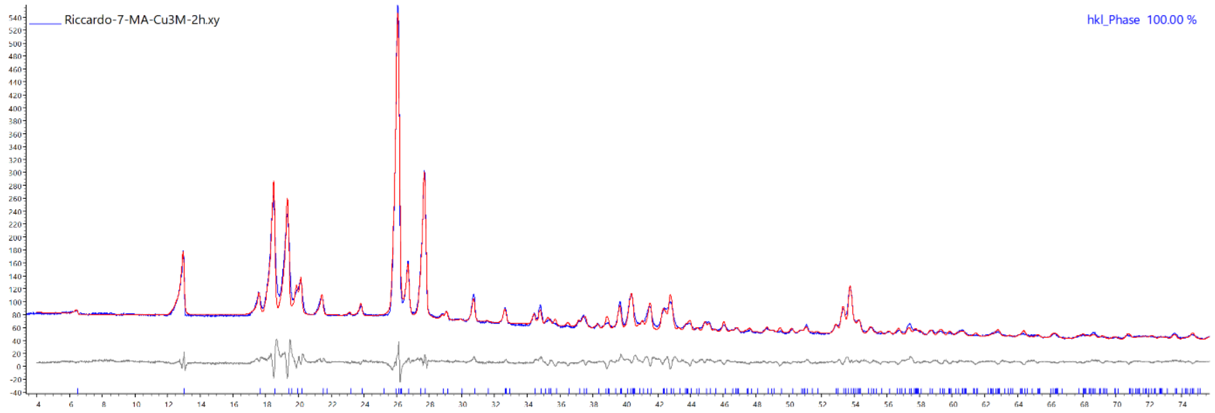

Figure S12. 7-methyladenine Rietveld refinement. The x-axis is given as 2θ in °, while the y-axis represents the intensity in arbitrary units. Radiation wavelength: 1.540596 Å (Cu/Kα1).

4. Diffraction patterns changes in function of temperature – 3ma

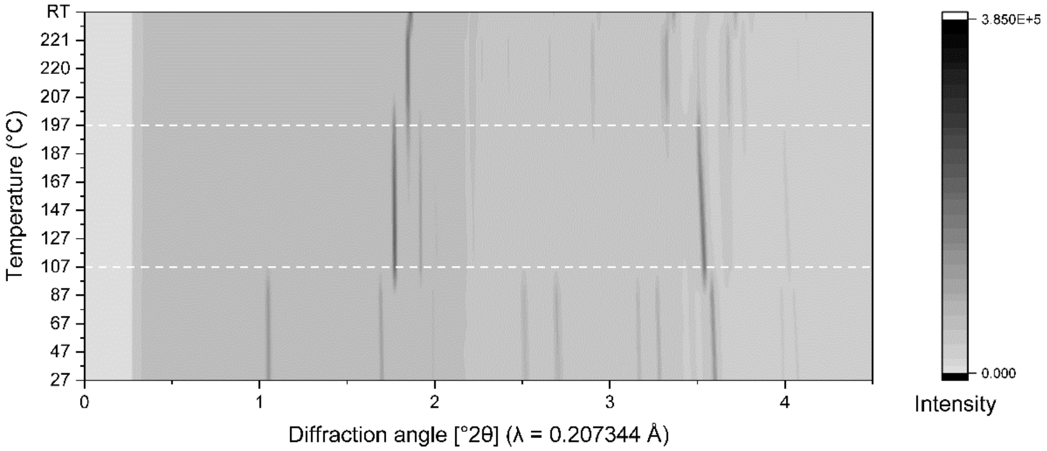

Figure S13. XRPD contour plot showing the structural changes of 3-methyladenine. Temperatures of water removal from the crystal structure (approximately at 100 °C) and formation of polymorph (approximately at 200 °C) are highlighted in the figure by white lines.

5. Raman spectra of methylated nucleobases

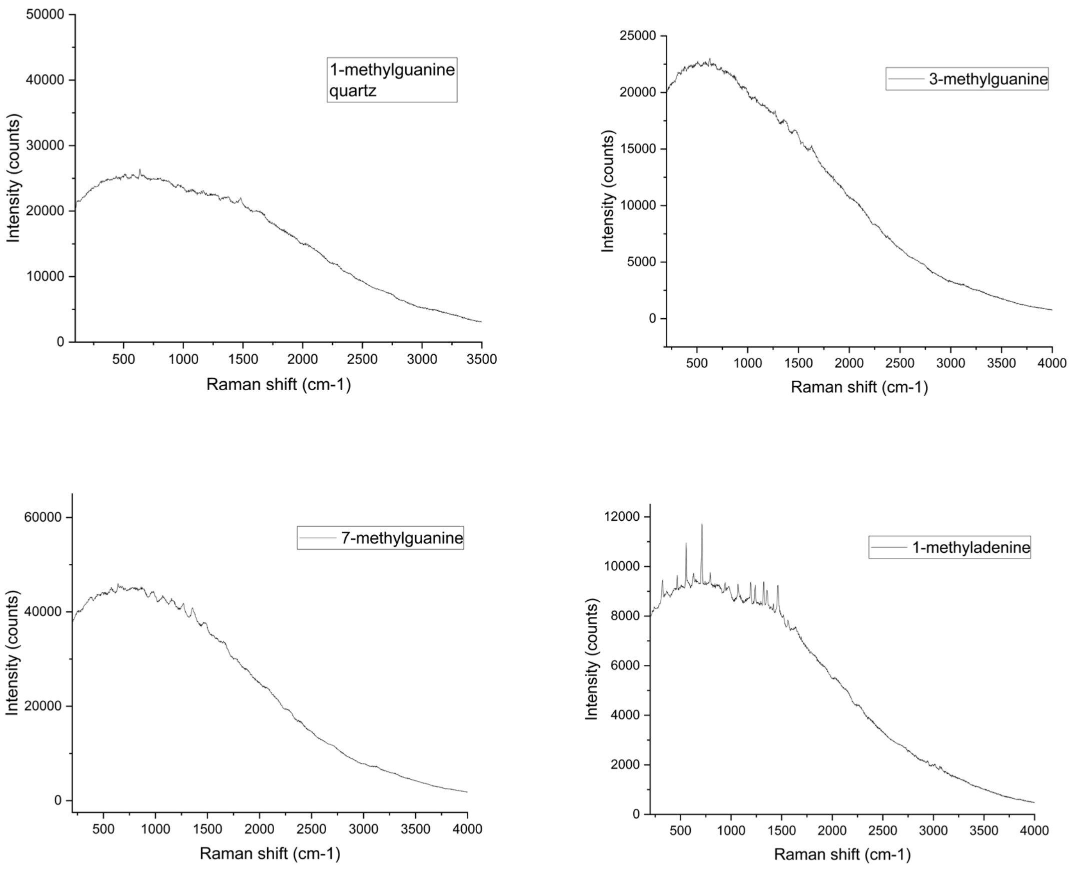

Figure S14. Raman spectra of nucleobases in quartz capillaries, excitation wavelength: 633 nm. 1-methylguanine (a). 3-methylguanine compound as purchased. It is inferred after DTA-TGA that it might be a different hydrate instead of a sesquihydrate. See main text for further information. 7-methylguanine (c). 1-methyladenine(d).

6. Tables with bond geometries determined of the investigated crystal structures

Table S1: Full H-bond geometries considered in this study for *N*-methylated guanines and adenines (Å, °).								
1-methylguanine								
D-H···A		d(D-H)		d(H···A)		d(D···A)		∠(D-H···A)
N2-H2···N8 (i)		0.88		1.98		2.844(2)		167
N5-H5A···O2 (ii)		0.88		2.11		2.893(2)		148
N5-H5B···N6 (ii)		0.88		2.13		2.822(2)		135
N7-H7···N3 (iii)		0.88		1.98		2.843(2)		168
N10-H10A···O1 (iv)		0.88		2.11		2.903(2)		149
N10-H10B···N1 (iv)		0.88		2.13		2.815(2)		134
C3_a-H3···O1 (i)		0.95		2.4		3.262(2)		150
C9-H9···O2 (iii)		0.95		2.39		3.257(2)		152

Symmetry codes: (i) 1-x, -1/2 + y, 1/2 - z; (ii) x, y, z; (iii) 1 - x, 1/2 + y, 3/2 - z; (iv) -1 + x, y, z.				
3-methylguanine sesquihydrate				
D-H···A	d(D-H)	d(H···A)	d(D···A)	∠(D-H···A)
O3-H3A···N8 (i)	0.883(17)	1.887(17)	2.7680(11)	174.7(16)
O3-H3B···O2 (ii)	0.883(16)	1.863(16)	2.7406(10)	172.1(15)
N4-H4···O1 (iii)	0.934(16)	2.540(17)	3.1075(11)	119.5(12)
N4-H4···N6 (iii)	0.934(16)	1.877(16)	2.8112(12)	179.3(19)
O4-H4A···N3 (iv)	0.891(16)	1.947(16)	2.8332(11)	173.5(15)
O4-H4B···O3 (i)	0.888(17)	1.904(17)	2.7868(12)	172.9(17)
N5-H5A···O5 (v)	0.905(14)	2.025(14)	2.8878(11)	158.9(13)
N5-H5B···O1 (vi)	0.916(15)	1.961(15)	2.8577(11)	165.8(13)
O5-H5C···O4 (v)	0.884(17)	1.793(17)	2.6764(11)	177.1(19)
O5-H5D···O3 (vii)	0.897(16)	1.875(16)	2.7694(11)	175.8(17)
N9-H9···O2 (vi)	0.944(16)	2.600(16)	3.1766(11)	119.9(12)
N9-H9···N1 (vi)	0.944(16)	1.833(16)	2.7763(12)	178.6(16)
N10-H10A···O5 (viii)	0.903(15)	2.061(15)	2.9342(12)	162.5(15)
N10-H10B···O2 (iii)	0.900(15)	2.012(15)	2.8798(12)	161.6(13)
Symmetry codes: (i) x, y, z; (ii) -1 + x, y, z; (iii) 2 - x, -y, 1 - z; (iv) 1 - x, 1/2 + y, 1/2 - z; (v) 1 - x, 1 - y, 1 - z; (vi) 2 - x, 1 - y, 1 - z; (vii) x, 1/2 - y, 1/2 + z; (viii) 1 - x, - y, 1 -z.				
3-methylguanine				
D-H···A	d(D-H)	d(H···A)	d(D···A)	∠(D-H···A)
N2-H1···O1 (i)	0.90(2)	1.99(2)	2.867(16)	164(2)
N2-H2···N5 (ii)	0.90(2)	3.30(2)	4.18(2)	166(2)
N4-H3···N1 (iii)	0.946(18)	1.832(18)	2.751(17)	163(18)
Symmetry codes: (i) 1 - x, 1/2 + y, 1/2 - z; (ii) ? - not reported by Platon - ; (iii) 1 - x, -1/2 + y, 1/2 - z;				
7-methylguanine				
D-H···A	d(D-H)	d(H···A)	d(D···A)	∠(D-H···A)
N2-H1···N5_4 (i)	1.0(4)	1.8(4)	2.7(3)	150(1)
N2_2-H1_2···N5_3 (ii)	0.9(3)	2.4(3)	3.35(18)	174(1)
N2-H2···O1_4 (iii)	0.9(3)	2.3(4)	3.2(2)	164(1)
N2_2-H2_2···O1_3 (ii)	0.9(3)	1.9(2)	2.8(2)	167(1)
N1_b-H3···N3_4 (i)	1.0(3)	1.9(3)	2.8(3)	151(1)
N1_2-H3_2···N3_3 (iv)	0.9(2)	2.1(2)	2.9(2)	156(1)
N2_3_g-H1_3···N5_2 (ii)	0.9(4)	2.5(3)	3.4(3)	169(1)
N2_3-H2_3···O1_2 (ii)	0.9(2)	1.8(2)	2.75(19)	176(1)
N1_3-H3_3···N3_2 (ii)	1.0(3)	2.1(3)	3.0(3)	161(17)
N2_4-H1_4···N5 (iii)	0.9(3)	1.9(3)	2.8(3)	164(1)
N2_4-H2_4···O1 (i)	0.9(3)	2.1(3)	3.0(2)	162(1)
N1_4-H3_4···N3 (v)	1.0(3)	2.0(3)	2.9(3)	159(19)
C5_2-H4_2···N4_4 (vi)	0.9(2)	2.5(2)	3.3(2)	144(1)

C6-H7···O1_4 (vii)	1.0(2)	2.5(2)	3.3(2)	135(18)
C5_3-H4_3···O1_4 (vi)	0.9(3)	2.5(2)	3.4(2)	161(16)
C6_4-H6_4···N5 (viii)	0.9(2)	2.5(2)	3.2(2)	141(17)
Symmetry codes: (i) 2 - x, 1 - y, -z; (ii) - 1 - x, -y, -1 - z; (iii) 1 - x, 1 - y, -z; (iv) -2 - x, -y, -1 - z; (v) 1 - x, 1 - y, -1 - z; (vi) -x, 1 - y, -1 - z; (vii) x, y, 1 + z; (viii) x, y, -1 + z.				
1-methyladenine				
D-H···A	d(D-H)	d(H···A)	d(D···A)	∠(D-H···A)
N5-H3···N3_4 (i)	1.01(16)	2.29(17)	3.26(16)	161(11)
N5_2-H3_2···N3_3 (ii)	1.02(16)	2.40(16)	3.39(14)	165(9)
N5-H4···N4	1.04(13)	1.90(14)	2.83(13)	147(10)
N5_2-H4_2···N4_2	1.03(14)	2.12(14)	2.85(14)	126(10)
N5_3-H3_3···N2 (iii)	1.03(14)	2.18(17)	3.14(17)	154(9)
N5_3-H4_3···N4_3	1.03(17)	1.92(16)	2.91(16)	161(10)
N5_4-H3_4···N2_2 (iv)	1.02(16)	2.7(2)	3.5(2)	137(11)
N5_4_j-H4_4···N4_4	1.02(17)	1.92(16)	2.88(16)	156(11)
C1-H1···N2_4 (v)	1.01(18)	2.44(19)	3.27(18)	140(15)
C5-H2···N3_4 (i)	1.0(2)	2.50(18)	3.45(18)	156(14)
C6-H5···N4	1.01(16)	2.54(16)	3.42(16)	146(12)
C6_2-H6_2···N5_2	1.01(16)	1.94(15)	2.51(16)	113(10)
C6-H7···N2_4 (vi)	1.01(17)	2.59(16)	3.54(17)	157(10)
C1_3-H1_3···N3 (vii)	1.0(2)	2.11(19)	3.13(19)	178(1)
C6_3-H5_3···N4_3	1.02(15)	2.43(16)	3.07(16)	121(10)
C1_4-H1_4···N3_2 (viii)	1.0(2)	2.05(19)	3.01(19)	156(12)
C5_4-H2_4···N2_2 (ix)	1.0(2)	2.46(19)	3.4(2)	151(12)
C6_4-H6_4···N4_4	1.00(17)	2.29(16)	3.19(16)	149(2)
C6_4-H7_4···N4_3 (x)	1.02(13)	2.50(13)	3.33(13)	140(11)
Symmetry codes: (i) -x; -1/2 + y, -z; (ii) -1 + x, 1 + y, -1 + z; (iii) x, -1 + y, 1 + z; (iv) ? - not reported by Platon - ; (v) 1 + x, y, -1 + z; (vi) 1 + x, y, z; (vii) 1 - x, -1/2 + y, -z; (viii) x, y, 1 + z; (ix) -1 - x, 1/2 + y, -z; (x) -1 + x, 1/2 + y, -z.				
3-methyladenine monohydrate				
D-H···A	d(D-H)	d(H···A)	d(D···A)	∠(D-H···A)
N5-H3···N3 (i)	1.009(7)	1.932(7)	2.883(8)	156.1(6)
N5-H4···N1 (ii)	1.030(8)	2.162(9)	3.102(9)	150.7(4)
O1_a-H8···O1 (iii)	0.950(6)	2.452(5)	2.908(6)	109.4(3)
O1-H9···O1 (iv)	0.950(6)	2.566(6)	2.908(6)	101.5(4)
C5-H2···N1 (v)	1.019(2)	2.55(11)	3.473(11)	150.5(8)
Symmetry codes: (i) -1 -x, 2 - y, -z; (ii) -1 -x, 1/2 + y, -1/2 - z; (iii) 1 - x, -1/2 + y, 1/2 - z; (iv) 2 - x, -1/2 + y, 1/2 - z; (v) x, 3/2 - y, 1/2 - z.				
3-methyladenine				
D-H···A	d(D-H)	d(H···A)	d(D···A)	∠(D-H···A)
N5-H3···N1 (i)	1.01(2)	2.14(4)	3.02(4)	144(2)
N5-H4···N3 (ii)	1.03(2)	2.00(3)	3.03(3)	173(2)
C6-H6···N4	1.01(3)	2.68(4)	3.25(4)	116(2)

Symmetry codes: (i) x, -3/2 - y, 1/2 + z; (ii) x, -3/2 - y, -1/2 + z.				
3-methyladenine polymorph				
D-H···A	d(D-H)	d(H···A)	d(D···A)	∠(D-H···A)
N5_b-H3···N1 (i)	1.01(10)	2.053(11)	2.990(11)	153.3(7)
N5-H4···N3 (ii)	1.03(11)	2.003(11)	2.968(12)	155.0(6)
C1-H1···N4 (iii)	1.02(13)	2.577(11)	3.551(12)	159.7(8)
Symmetry codes: (i) -1/2 - x, -1/2 + y, 7/4 - z; (ii) -3/2 - x, 1/2 + y, 7/4 - z; (iii) -1 + y, 1 + x, 2 - z.				
7-methyladenine				
D-H···A	d(D-H)	d(H···A)	d(D···A)	∠(D-H···A)
N5-H3···N2 (i)	1.011(17)	2.087(16)	2.949(16)	141.8(13)
N5-H4···N1 (ii)	1.031(19)	2.083(19)	3.112(19)	176.8(11)
C1_a-H1···N1 (iii)	1.02(2)	2.49(2)	3.50(2)	176.6(15)
C5-H2···N4 (iv)	1.02(3)	2.43(2)	3.37(2)	152.7(15)
C6-H6···N2 (i)	1.012(17)	2.571(18)	3.271(18)	126.0(13)
C6-H7···N4 (v)	1.013(19)	2.568(16)	3.388(17)	137.7(11)
Symmetry codes: (i) x, -1 + y, -1 + z; (ii) 1/2 - x, -1 - y, -1/2 + z; (iii) 1/2 - x, -1 - y, 1/2 + z; (iv) -x, -1/2 + y, 3/2 - z; (v) x, y, -1 + z.				

Table S2: Full stacking geometrical parameters considered in this study for N-methylated guanines and adenines (Å, °).

1-methylguanine									
Centroids interaction*	Cg···Cg distance	Cg(I)-Perp	Cg(J)-Perp	α angle	β angle	Slippage	Symmetry code	N-rings contacts	Rot. angle and config.
Cg1···Cg1	4.2528(11)	3.3025(8)	3.3025(8)	0.99(11)	39.1	2.679	2-x,1-y,1-z	5_5	
Cg1···Cg4	4.1950(11)	3.2933(8)	3.1875(8)	3.33(11)	40.6	2.727	1-x, 1-y, 1-z	5_5	
Cg4···Cg4	4.7918(11)	3.2049(8)	3.1849(8)	3.04(11)	38.3	3.58	x, 3/2-y, -1/2+z	5_5	
Cg2···Cg2	3.7440(10)	3.306(7)	3.3060(7)	0.03(8)	28	1.757	2-x, 1-y, 1-z	6_6	
Cg2···Cg6	4.3519(11)	3.3002(7)	3.2112(7)	1.77(8)	42.4	2.937	1-x, 1-y, 1-z	6_6	
Cg6···Cg6	5.8593(10)	3.2229(7)	3.2574(7)	0.58(8)	56.2	4.87	x, 3/2-y, -1/2+z	6_6	
Cg3···Cg3	3.4595(9)	3.3091(6)	3.3138(7)	0.27(7)	16.7	0.993	2-x, 1-y, 1-z	9_9	180.0(1), trans
Cg3···Cg7	4.0379(8)	3.2801(6)	3.1858(6)	2.30(6)	37.9	2.481	1-x, 1-y, 1-z	9_9	82.6(2), trans
Cg7···Cg7	5.2103(8)	3.2316(6)	3.2021(6)	0.81(6)	52.1	4.11	x, 3/2-y, -1/2+z	9_9	79.8(3), trans
*Centroids defined for aromatic rings as follows: imidazole rings: Cg1 = N1C2C4N2C3; Cg4 = N6C8C10N7C9; pyrimidine rings: Cg2 = N3C4C2C1N4C5; Cg6 = N9C7C8C10N8C11; pyrimidine+imidazole rings Cg3 = N1C2C1N4C5N3C4N2C3; Cg7 = N6C8C7N9C11N8C10N7C9									
3-methylguanine sesquihydrate									
Centroids interaction*	Cg···Cg distance	Cg(I)-Perp	Cg(J)-Perp	α angle	β angle	Slippage	Symmetry code	N-rings contacts	Rot. angle and config.
Cg1···Cg4	3.4304(6)	3.2546(4)	3.2417(4)	1.75(6)	19.1	1.122	x, y, z	5_5	

		5.6912(6)	3.2546(4)	3.2417(4)	2.13(6)	57.1	4.777	x, 1/2-y, 1/2+z	5_5	
Cg2···Cg5		4.3927(5)	3.2713(4)	3.3193(4)	4.34(4)	40.9	2.877	x, y, z	6_6	
		5.7554(5)	3.3922(4)	3.0367(4)	5.82(4)	58.2	4.889	x, 1/2-y, 1/2+z	6_6	
Cg3···Cg6		3.4578(4)	3.2409(3)	3.2575(3)	3.54(3)	19.6	1.16	x, y, z	9_9	159.85(8), cis
		5.7300(4)	3.3377(3)	3.0500(3)	4.49(3)	57.8	4.851	x, 1/2-y, 1/2+z	9_9	10.6(2), trans
*Centroids defined for aromatic rings as follows: imidazole rings: Cg1 = N8C3C4N9C5; Cg4 = N3C9C10N4C11; pyrimidine rings: Cg2 = N6C1C4C3N7C2; Cg5 = N1C7C10C9N2C8; pyrimidine+imidazole rings: Cg3 = N6C1C3N9C5N8C3N7C2; Cg6 = N1C7C10N4C11N3C9N2C8.										
3-methylguanine										
Centroids interaction*		Cg···Cg distance	Cg(I)-Perp	Cg(J)-Perp	α angle	β angle	Slippage	Symmetry code	N-rings contacts	Rot. angle and config.
Cg1···Cg1		3.937(15)	3.302(11)	3.302(11)	0	33	2.144	1-x, -y, 1-z	5_5	
		3.611(14)	3.290(11)	3.291(11)	0	24.3	1.487	2-x, -y, 1-z	5_5	
Cg2···Cg2		4.335(13)	3.305(11)	3.305(11)	0	40.3	2.806	1-x, -y, 1-z	6_6	
		4.351(13)	3.290(11)	3.290(11)	0	40.9	2.847	2-x, -y, 1-z	6_6	
Cg3···Cg3		3.604(11)	3.306(8)	3.306(8)	0.0(7)	23.4	1.434	1-x, -y, 1-z	9_9	180(3), trans
		3.465(11)	3.288(8)	3.288(8)	0.0(7)	18.4	1.84	2-x, -y, 1-z	9_9	180(2), trans
*Centroids defined for aromatic rings as follows: imidazole rings: Cg1 = N4C3C2N5C5; pyrimidine rings: Cg2 = N1C1N3C2C3C4; pyrimidine+imidazole rings: Cg3 = N1C1N3C2N5C5N4C3C4.										
7-methylguanine										
Centroids interaction*		Cg···Cg distance	Cg(I)-Perp	Cg(J)-Perp	α angle	β angle	Slippage	Symmetry code	N-rings contacts	Rot. angle and config.
Cg1···Cg1		5.07(11)	3.28(10)	3.28(10)	0	49.6	3.863	-2-x, -y, -1-z	5_5	
Cg1···Cg13		4.56(16)	3.34(10)	3.44(10)	4	41	2.989	x, y, z	5_5	
Cg5···Cg14		5.16(14)	3.57(10)	3.43(10)	4	48.3	3.855	x, 1+y, z	6_6	
		4.87(14)	3.36(10)	3.21(10)	4	48.6	3.654	x, 2+y, z	6_6	
Cg5···Cg5		4.06(15)	3.25(10)	3.26(10)	0	36.7	2.429	-2-x, 2-y, -1-z	6_6	
Cg15···Cg16**		5.38	3.196	3.365	3.99	53.55	4.328	x, 2+y, z	9_9	35(21), trans
Cg15···Cg15**		4.003	3.265	3.265	0	35.35	2.31	-2-x, 2-y, -1-z	9_9	180(42), trans
*Centroids defined for aromatic rings as follows: imidazole rings: Cg1 = N4_3C3_3C2_3N5_3C5_3; Cg13 = N4_2C3_2C2_2N5_2C5_2; pyrimidine rings: Cg5 = N3_3C2_3C3_3C4_3N1_3C1_3; Cg14 = N1_2C1_2N3_2C2_2C3_2C4_2; pyrimidine+imidazole rings: Cg15 = C2_3N5_3C5_3N4_3C3_3N3_3C2_3C3_3C4_3N1_3C1_3; Cg16 = C2_2N5_2C5_2N4_2C3_2N3_2C2_2C3_2C4_2N1_2C1_2. **Centroids selected and values calculated from Mercury.										
1-methyladenine										
Centroids interaction*		Cg···Cg distance	Cg(I)-Perp	Cg(J)-Perp	α angle	β angle	Slippage	Symmetry code	N-rings contacts	Rot. angle and config.
Cg5···Cg1		5.14(8)	3.67(5)	3.56(5)	14	46.1	3.705	1+x, y, z	5_5	
Cg10···Cg7		4.84(9)	3.69(5)	3.35(5)	6	46.2	3.493	-1+x, 1+y, z	5_5	
Cg6···Cg3		4.52(7)	3.32(5)	3.97(5)	15	28.9	2.182	1+x, y, z	6_6	
Cg11···Cg9		3.65(6)	3.49(4)	3.55(5)	6	13.6	0.857	-1+x, 1+y, z	6_6	
Cg12···Cg13**		4.341	3.476	3.792	14.58	36.8	2.6	1+x, y, z	9_9	170(13), trans
Cg14···Cg15**		3.673	3.465	3.578	6.37	19.37	1.219	-1+x, 1+y, z	9_9	176(19), trans

*Centroids defined for aromatic rings as follows: imidazole rings: Cg1 = N3_2C3_2C2_2N4_2C5_2; Cg5 = N3C3C2N4C5; Cg10 = N3_4C3_4C2_4N4_4C5_4; Cg7 = N3_3C3_3C2_3N4_3C5_3; pyrimidine rings: Cg6 = N1C1N2C2C3C4; Cg3 = N1_2C1_2N2_2C2_2C3_2C4_2; Cg11 = N1_4C1_4N2_4C2_4C3_4C4_4; Cg9 = N1_3C1_3N2_3C2_3C3_3C4_3; pyrimidine+imidazole rings: Cg12 = N3C3C2N4C5N1C1N2C4; Cg13 = N3_2C3_2C2_2N4_2C5_2N1_2C1_2N2_2C4_2; Cg14 = N3_3C3_3C2_3N4_3C5_3N1_3C1_3N2_3C4_3; Cg15 = N3_4C3_4C2_4N4_4C5_4N1_4C1_4N2_4C4_4. **Centroids selected and values calculated from Mercury.

3-methyladenine monohydrate										
Centroids interaction*		Cg···Cg distance	Cg(I)-Perp	Cg(J)-Perp	α angle	β angle	Slippage	Symmetry code	N-rings contacts	Rot. angle and config.
Cg1···Cg1		5.01(5)	3.399(4)	3.399(4)	0(5)	47.3	3.681	x, -1+y, z	5_5	
		5.010(5)	3.399(4)	3.399(4)	0(5)	47.3	3.681	x, 1+y, z	5_5	
		4.900(5)	3.264(4)	3.264(4)	0(5)	48.2	3.654	-1 -x, 1 - y, -z	5_5	
Cg2···Cg2		5.009(5)	3.453(3)	3.453(3)	0(4)	46.4	3.629	x, -1+y, z	6_6	
		5.010(5)	3.453(3)	3.453(3)	0(4)	46.4	3.63	x, 1+y, z	6_6	
		4.945(5)	3.255(3)	3.255(3)	0(4)	48.8	3.722	-1 -x, 1 - y, -z	6_6	
Cg3···Cg3		5.010(4)	3.444(3)	3.444(3)	0(2)	46.6	3.638	x, -1+y, z	9_9	0.0(7), cis
		5.010(4)	3.444(3)	3.444(3)	0(2)	46.6	3.638	x, 1+y, z	9_9	0.0(7), cis
		4.485(4)	3.268(3)	3.268(3)	0(2)	43.2	3.072	-1 -x, 1 - y, -z	9_9	180.0(9), trans

*Centroids defined for aromatic rings as follows: imidazole rings: Cg1 = N3C3C2N4C5; pyrimidine rings: Cg2 = N1C1N2C2C3C4; pyrimidine+imidazole rings: Cg3 = N1C1N2C2N4C5N3C3C4.

3-methyladenine										
Centroids interaction*		Cg···Cg distance	Cg(I)-Perp	Cg(J)-Perp	α angle	β angle	Slippage	Symmetry code	N-rings contacts	Rot. angle and config.
Cg1···Cg1		4.37(3)	3.207(18)	3.209(18)	0	42.7	2.962	1-x, -1-y, -2-z	5_5	
		4.24(3)	3.661(18)	3.659(18)	0	30.4	2.144	2-x, -1-y, -2-z	5_5	
Cg2···Cg2		3.56(2)	3.223(14)	3.222(14)	0	21.2	1.253	1-x, -1-y, -2-z	6_6	
		4.40(2)	3.638(14)	3.638(14)	0	34.3	2.477	2-x, -1-y, -2-z	6_6	
Cg3···Cg3		3.291(16)	3.223(12)	3.223(12)	0	11.7	0.667	1-x, -1-y, -2-z	9_9	180(4), trans
		3.814(16)	3.640(12)	3.641(12)	0	17.4	1.137	2-x, -1-y, -2-z	9_9	180(5), trans

*Centroids defined for aromatic rings as follows: imidazole rings: Cg1 = N3C3C2N4C5; pyrimidine rings: Cg2 = N1C1N2C2C3C4; pyrimidine+imidazole rings: Cg3 = N1C1N2C2N4C5N3C3C4.

3-methyladenine polymorph										
Centroids interaction*		Cg···Cg distance	Cg(I)-Perp	Cg(J)-Perp	α angle	β angle	Slippage	Symmetry code	N-rings contacts	Rot. angle and config.
Cg2···Cg2		3.574(5)	3.443(4)	3.443(4)	0.3(6)	15.5	0.958	-2+y, 2+x, 2-z	5_5	
		4.802(7)	1.443(4)	4.049(4)	40.5(6)	32.5		-3/2-x, -1/2+y, 7/4-z	5_5	
Cg4···Cg4		3.483(6)	3.448(4)	3.448(4)	1.4(5)	8.2	0.496	-2+y, 2+x, 2-z	6_6	
Cg5···Cg5		3.449(4)	3.446(3)	3.446(3)	0.6(3)	2.4	0.147	-2+y, 2+x, 2-z	9_9	22(1), trans
		5.442(5)	2.167(3)	4.775(3)	40.5(3)	28.7		-3/2-x, -1/2+y, 7/4-z	9_9	27(1), trans

*Centroids defined for aromatic rings as follows: imidazole rings: Cg2 = C2C3N3C5N4; pyrimidine rings: Cg4 = N2C1N1C4C3C2; pyrimidine+imidazole rings: Cg5 = N2C1N1C4C3N3C5N4C2.

7-methyladenine										
Centroids interaction*		Cg···Cg distance	Cg(I)-Perp	Cg(J)-Perp	α angle	β angle	Slippage	Symmetry code	N-rings contacts	Rot. angle and config.
Cg1···Cg1		5.102(9)	3.454(6)	3.454(6)	0(8)	47.4	3.755	x, -1+y, z		
		4.636(8)	3.406(6)	3.406(6)	0(8)	42.7	3.145	x, y, -1+z		
Cg2···Cg2		5.102(8)	3.432(6)	3.433(6)	0(7)	47.7	3.775	x, -1+y, z		
		4.635(8)	3.426(6)	3.425(6)	0(7)	42.4	3.122	x, y, -1+z		
Cg3···Cg3		5.102(7)	3.441(4)	3.441(4)	0(4)	47.6	3.766	x, -1+y, z	9_9	0(1), cis
		4.636(6)	3.417(4)	3.417(4)	0(4)	47.6	3.766	x, y, -1+z	9_9	0(2), cis

*Centroids defined for aromatic rings as follows: imidazole rings: Cg1 = N3C3C2N4C5; pyrimidine rings: Cg2 = N1C1N2C2C3C4; pyrimidine+imidazole rings: Cg3 = N1C1N2C2N4C5N3C3C4.
